# Visible-Light-Induced C–C Coupling Reaction to Synthesize Bipyridine From 3-Cyano-1,4-Dihydropyridines

**DOI:** 10.3389/fchem.2019.00940

**Published:** 2020-01-17

**Authors:** Shijun Chen, Qidi Zhong, Hao Zhu, Chunyan Liu, Pengyu Zhuang, Wuji Sun

**Affiliations:** ^1^School of Pharmacy, North China University of Science and Technology, Tangshan, China; ^2^School of Public Health, North China University of Science and Technology, Tangshan, China

**Keywords:** photocatalytic, C–C coupling, pyridines, radical reactions, 1,4-dihydropyridine

## Abstract

A concise and efficient photocatalytic C–C coupling of 1-benzyl-3-cyano-1, 4-dihydropyridine for synthesis of 1,1′-dibenzyl-3, 3′-dicyano-1,1′,4,4′-tetrahydro-4, 4′-bipyridine is described. The reporter system provides a novel technique that facilitates synthesis of C–C coupling derivatives without addition of transition metals and oxidants or other additives. A plausible synthetic pathway is proposed, and the coupling product was characterized via nuclear magnetic resonance spectroscopy (^1^H and ^13^C NMR), high-resolution electrospray ionization mass spectrometry (ESI-HRMS) and X-ray analyses.

## Introduction

**Figure 1:**
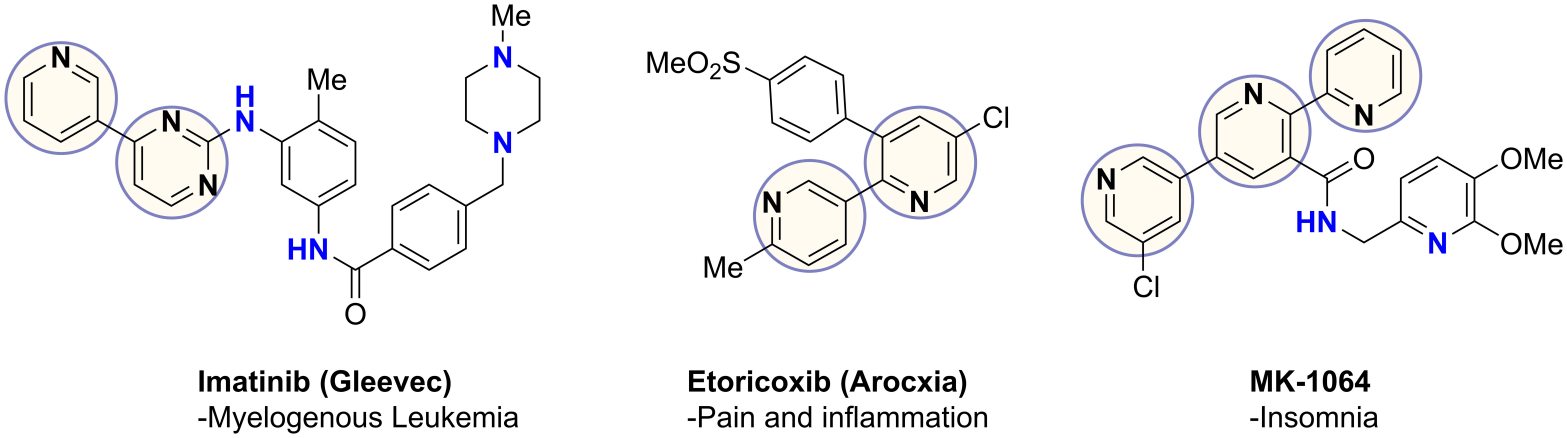
Important heterobiaryl containing drugs.

Coupling of pyridines and diazines results in the generation of heterobiaryls, a privileged pharmacophore found in commercial drugs as well as numerous therapeutic candidates, such as the examples in Figure 1 (Capdeville et al., 2002; Martina et al., 2005; Roecker et al., 2014). These heterocycles often play a key role in drug-receptor binding and confer other important properties, such as net polarity, aqueous solubility, and resistance to oxidative metabolism (Hilton et al., 2018; Zhang et al., 2019). The most common aryl-aryl coupling is achieved through metal-catalyzed cross-coupling reactions (Tahsini et al., 2019). C–C coupling reactions have become fundamental routes in natural product synthesis, materials science, biological-, medicinal-, and supramolecular chemistry, in addition to catalysis, coordination chemistry and polymer synthesis (Cocuzza et al., 1999; Akihiro et al., 2007; Heravi and Hashemi, 2012).

While C–C bond generation methods are effective reliable, these reactions require pre-activation steps, such as halogenation or metallization of reactants, thereby increasing the number of reaction steps and chemical reagents, and reducing the efficiency of synthesis.

Photocatalysis has developed significantly over the past decade, making possible synthetic transformations that were previously impossible (Ravelli et al., 2016; Romero and Nicewicz, 2016; She et al., 2018). The photochemical reaction does not require additional additives or reagents, thus embodying the concept of green chemistry (Norbert, 2008; Fan et al., 2017). To date, photochemical reactions have been applied to various fields of organic chemistry as eco-friendly and efficient synthesis method, including metal catalysis (Condie et al., 2010), green organic synthesis (Yu et al., 2018), total synthesis (Reddy and Rawal, 2000) and asymmetric synthesis (Richard et al., 2015) of compounds. Photosensitive 1,4-dihydropyridine is often used as a photoreactive substrate for various reactions under light-induced conditions, such as photoinduced rearrangement (Zhong et al., 2017), aromatization (Memarian and Mirjafari, 2005), and [2+2] photocycloaddition reactions (Eisner et al., 1970; Hilgeroth et al., 2000; Hilgeroth and Baumeister, 2001).

**Scheme 1:**
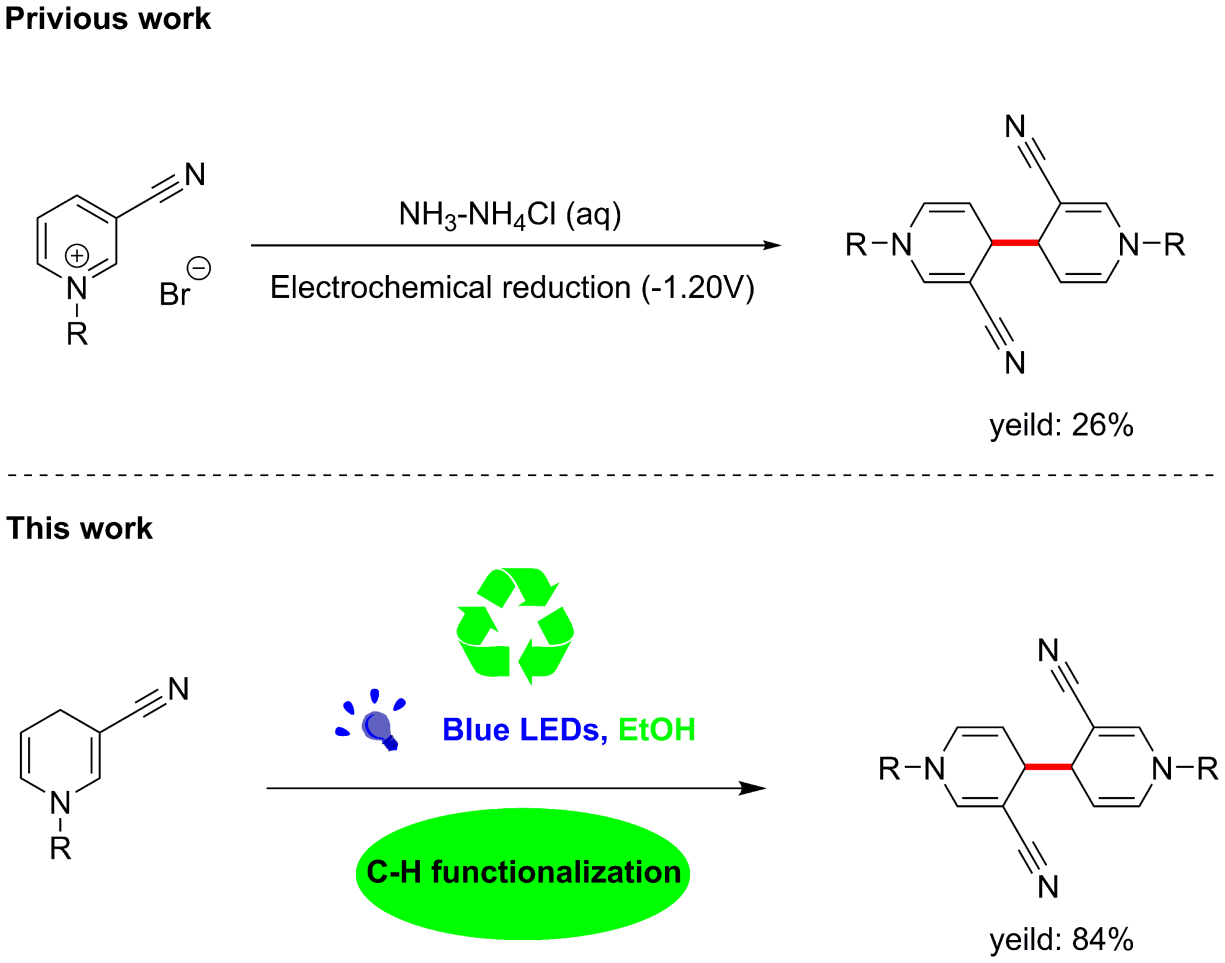
C–C coupling reaction of visible-light-catalyzed 1,4-dihydropyri-dine derivative.

Unexpectedly, the photoreaction product of 1,4-dihydropyridine we obtained was distinct from that reported previously. In the absence of electrochemical reduction (Carelli et al., 1998), photocatalysis can directly promote activation of sp3 bonds to form a coupled product form a coupled product (Scheme 1).

Based on earlier literature and our research on visible-light, we have developed a novel method for the construction of C–C bonds via visible light catalysis using 3-cyano-1,4-dihydropyridine as the light substrate and ethanol as solvent (Scheme 1). The visible-light-activated C-H bond is used for selective formation of new C–C bonds. This method has a number of advantages: (1) the reaction is carried out with no requirement for catalyst and transition metal, (2) the solvent is environmentally friendly, non-toxic and easy to use, and (3) reaction specificity and conversion rates are high, with few by-products.

## Materials and Methods

### General Information

Unless otherwise specified, all commercial reagents and solvents were used without further purification. Melting points were uncorrected and determined in open capillary tubes with WRX-4 micro melting point apparatus (China). Mass spectra (ESI-HRMS) were recorded on an Agilent Accurate-Mass Q-TOF LC/MS 6520 instrument. ^1^H NMR and ^13^C NMR spectra were recorded at 400 and 500 MHz using deuterated CDCl_3_ or DMSO-d_6_ solvent. Chemical shifts were expressed as parts per million (δ) relative to tetramethylsilane (TMS). Data are presented as follows order: chemical shift (δ) in ppm whereby; multiplicities are indicated as s (singlet), d (doublet), t (triplet), q (quartet), dd (doublet of doublets), td (triplet of doublets), tt (triplet of triplets), ddd (doublet of doublet of doublets), m (multiplet), qui (quint), sext (sextet), h(hept); coupling constants (*J*) in Hertz (Hz). Single-crystal X-ray diffraction was performed on a Bruker APEX-II instrument. Silica gel (200–300 mesh) for column chromatography and silica GF254 for TLC were obtained from Qingdao Marine Chemical Company (China).

### Preparation of Starting Materials

Substrates 1 (1a−1t) were synthesized according to documented literature (Paul et al., 2013), with slight modifications. In brief, benzyl bromide (40 mmol) was added to 3-cyanopyridine (4.16 g, 40 mmol) using acetonitrile (40 mL) as the solvent. The mixture was reacted for 7 h, and diethyl ether was added after cooling to precipitate the product. After filtering, the mixture was rinsed with ether three times (3× 10 mL) and the bromide salt obtained as a powder. Under nitrogen atmosphere, bromide salt (10.9 mmol) was dissolved in H_2_O (60 mL) and mixed with NaHCO_3_ (3.06 g, 36.4 mmol). Sodium dithionite (7.59 g, 43.6 mmol) was added slowly in stages and the reaction mixture stirred at room temperature for 1–3 h in the dark, during which time the solution was observed as a yellow precipitate. The solid was filtered, and washed with pure water (3× 10 mL) to generate product **1a−1t** as a yellow powder (Supplementary Material).

### General Procedure for Synthesis of Compounds 2a−2t

1a−1t (5 mmol) was dissolved in 25 mL absolute ethanol and placed in a three-necked quartz round bottomed flask. Argon-protection LED with a wavelength of 410 nm was used as the light source. After 3 days, the solvent was removed under reduced pressure and the crude reaction mixture was directly charged on silica gel and purified via column chromatography (petroleum ether/ethyl acetate rations of 4:1–5:1) to generate the corresponding 2a−2t product.

### Characterization of Products 2a

Synthesized according to the general procedure; Mp = 145–146°C (from CH_3_OH); ^1^H NMR (400 MHz, DMSO-d_6_): δ 7.40–7.26 (m, 12H), 6.08 (dd, *J* = 8, 1.2 Hz, 2H), 4.58 (dd, *J* = 8, 3.6 Hz, 2H), 4.42 (s, 4H), 3.19 (d, *J* = 3.6 Hz, 2H) ppm; ^13^C NMR (100 MHz, DMSO-d_6_) δ 145.6, 137.9, 130.9, 129.0, 128.0, 127.8, 121.3, 101.7, 77.3, 56.4 ppm; HRMS (ESI): C_26_H_22_N_4_ [M+H]^+^: 391.1878; found: 391.1897.

### Characterization of Products 2b

Synthesized according to the general procedure; Mp = 182–183°C (from CH_3_OH); ^1^H NMR (500 MHz, CDCl_3_): δ 7.28 (d, *J* = 8, 2H), 7.30–7.26 (m, 2H), 7.22–7.17 (m, 4H), 6.74(d, *J* = 1.5, 2H), 5.9 (dd, *J* = 8.5, 1.5 Hz, 2H), 4.77 (dd, *J* = 8 3.5 Hz, 2H), 4.36(s, 4H), 3.44 (d, *J* = 3.5 Hz, 2H) ppm; ^13^C NMR (125 MHz, CDCl_3_) δ 144.2, 135.3, 133.3, 129.9, 129.8, 129.3, 127.8, 123.2, 120.6, 103.0, 79.5, 57.4, 39.6 ppm; HRMS (ESI): C_26_H_20_Br_2_N_4_ [M+H]^+^: 547.0088; found: 547.0107.

### Characterization of Products 2c

Synthesized according to the general procedure; Mp = 174–175°C (from CH_3_OH); ^1^H NMR (400 MHz, DMSO-d_6_): δ 7.46–7.43 (m, 2H), 7.33–7.27 (m, 8H), 6.13 (d, *J* = 8.4 Hz, 2H), 4.66 (dd, *J* = 8, 3.6 Hz, 2H), 4.51 (s, 4H), 3.22 (d, *J* = 3.6 Hz, 2H) ppm; ^13^C NMR (100 MHz, DMSO-d_6_) δ 145.8, 135.3, 132.5, 130.9, 129.9, 129.7, 129.5, 127.9, 121.1, 102.0, 77.7, 54.2, 39.6 ppm; HRMS (ESI): C_26_H_20_Cl_2_N_4_ [M+H]^+^: 459.1098; found: 459.1088.

### Characterization of Products 2d

Synthesized according to the general procedure; Mp = 135–136°C (from CH_3_OH); ^1^H NMR (400 MHz, DMSO-d_6_): δ 7.39–7.34 (m, 2H), 7.29 (d, *J* = 1.5 Hz, 2H), 7.11–7.04 (m, 6H), 6.06 (dd, *J* = 8, 1.2 Hz, 2H), 4.51 (dd, *J* = 8, 3.6 Hz, 2H), 4.41 (s, 4H), 3.17 (d, *J* = 3.2 Hz, 2H) ppm; ^13^C NMR (100 MHz, DMSO-d_6_) δ 163.9, 161.4, 145.6, 145.5, 140.9, 131.1, 131.0, 130.8, 130.6, 123.7, 121.2, 114.8, 114.6, 114.4, 102.0, 101.8, 77.7, 55.8, 39.5 ppm; HRMS (ESI): C_26_H_20_F_2_N_4_ [M+H]^+^: 427.1689; found: 427.1676.

### Characterization of Products 2e

Synthesized according to the general procedure; Mp = 155–157°C (from CH_3_OH); ^1^H NMR (400 MHz, DMSO-d_6_): δ 7.20–7.12 (m, 10H), 6.12 (dd, *J* = 8.4, 1.2 Hz, 2H), 4.64 (dd, *J* = 8, 3.6 Hz, 2H), 4.42 (s, 4H), 3.25 (d, *J* = 3.2 Hz, 2H), 2.21 (s, 6H) ppm; ^13^C NMR (100 MHz, DMSO-d_6_) δ 145.7, 136.2, 135.9, 131.1, 130.7, 127.9, 127.8, 126.5, 121.3, 101.7, 77.4, 54.6, 39.7, 19.2 ppm; HRMS (ESI): C_28_H_26_N_4_ [M+H]^+^: 419.2191; found: 419.2188.

### Characterization of Products 2f

Synthesized according to the general procedure; Mp = 122–124°C (from CH_3_OH); ^1^H NMR (500 MHz, CDCl_3_): δ 7.24–7.21 (t, *J* = 8 Hz 2H), 6.80 (dd, *J* = 8, 1.5 Hz, 2H), 6.72 (t, *J* = 7.5 Hz, 6H), 5.91 (dd, *J* = 8 1 Hz, 2H), 4.72 (dd, *J* = 8 3.5 Hz, 2H), 4.24 (s, 4H) 3.76 (s, 6H), 3.39 (s, 2H) ppm; ^13^C NMR (125 MHz, CDCl_3_) δ 160.1, 144.2, 137.9, 130.2, 130.0, 120.8, 119.4, 113.4, 112.8, 102.7, 79.1, 57.4, 55.3, 39.6 ppm; HRMS (ESI): C_26_H_20_Br_2_N_4_ [M+H]^+^: 547.0088; found: 547.0083.

### Characterization of Products 2g

Synthesized according to the general procedure; Mp = 134–136°C (from CH_3_OH); ^1^H NMR (500 MHz, CDCl_3_): δ 7.43–7.08 (m, 8H), 6.72 (d, *J* = 1.5 Hz, 2H), 6.58 (dd, *J* = 8 1.5 Hz, 2H), 4.75 (dd, *J* = 8 3.5 Hz, 2H), 4.28 (d, *J* = 2 Hz, 4H), 3.41 (d, *J* = 3.5 2H) ppm; ^13^C NMR (125 MHz, CDCl_3_) δ 144.7, 144.0, 138.7, 131.3, 130.5, 130.2, 130.1, 129.7, 126.0, 125.8, 123.0, 120.5, 103.0, 102.9, 79.6, 57.1, 57.0, 40.7, 39.5 ppm; HRMS (ESI): C_26_H_20_Br_2_N_4_ [M+H]^+^: 547.0088; found: 547.0083.

### Characterization of Products 2h

Synthesized according to the general procedure; Mp = 128–130°C (from CH_3_OH); ^1^H NMR (500 MHz, CDCl_3_): δ 7.26–7.22(m, 4H), 7.15 (s, 2H), 7.05 (d, *J* = 4 Hz, 2H), 6.72(s, 2H), 5.95 (d, *J* = 8 Hz, 2H), 4.76 (dd, *J* = 8 3 Hz, 2H), 4.28 (d, *J* = 2.5 Hz, 4H), 3.42 (d, *J* = 3.5 Hz, 2H) ppm; ^13^C NMR (125 MHz, CDCl_3_) δ 144.0, 138.5, 134.8, 130.1, 128.3, 127.1, 125.3, 102.4, 103.0, 79.7, 77.2, 57.0, 39.5 ppm; HRMS (ESI): C_26_H_20_Cl_2_N_4_ [M+H]^+^: 459.1098; found: 459.1097.

### Characterization of Products 2i

Synthesized according to the general procedure; Mp = 129–130°C (from CH_3_OH); ^1^H NMR (500 MHz, CDCl_3_): δ 7.34–7.30 (m, 2H), 7.18–7.05 (m, 6H), 6.72 (d, *J* = 1 Hz, 2H), 5.82 (dd, *J* = 8 1.5 Hz, 2H), 4.65 (dd, *J* = 8 3.5 Hz, 2H), 4.29 (s, 4H), 3.34 (d, *J* = 3.5 2H) ppm; ^13^C NMR (125 MHz, CDCl_3_) δ 161.7, 159.7, 144.0, 130.2, 129.6, 124.5, 123.4, 123.3, 120.6, 115.9, 115.7, 102.7, 79.3, 77.2, 51.5, 39.5 ppm; HRMS (ESI): C_26_H_20_F_2_N_4_ [M+H]^+^: 427.1689; found: 427.1683.

### Characterization of Products 2j

Synthesized according to the general procedure; Mp = 121–123°C (from CH_3_OH); ^1^H NMR (400 MHz, DMSO-d_6_): δ 7.25–7.13 (m, 10H), 6.01 (dd, *J* = 8.4, 1.2 Hz, 2H), 4.53 (dd, *J* = 8, 3.2 Hz, 2H), 4.32 (s, 4H), 3.25 (d, *J* = 3.6 Hz, 2H), 2.27 (S, 6H) ppm; ^13^C NMR (100 MHz, DMSO-d_6_) δ 145.5, 137.2, 134.8, 130.8, 129.5, 127.8, 121.3, 101.7, 77.2, 56.2, 39.6, 21.1 ppm; HRMS (ESI): C_28_H_26_N_4_ [M+H]^+^: 419.2191; found: 419.2205.

### Characterization of Products 2k

Synthesized according to the general procedure; Mp = 155–156°C (from CH_3_OH); ^1^H NMR (500 MHz, CDCl_3_): δ 7.45 (d, *J* = 8.5 Hz, 4H), 7.04 (d, *J* = 8.5 Hz, 4H), 6.69 (d, *J* = 1.5 Hz, 2H), 5.83 (dd, *J* = 8.5 1.5 Hz, 2H), 4.71 (dd, *J* = 8 4 Hz, 2H), 4.23 (s, 4H), 3.39 (d, *J* = 4 Hz, 2H) ppm; ^13^C NMR (125 MHz, CDCl_3_) δ 144.1, 135.1, 132.1, 129.8, 128.9, 122.2, 120.5, 103.2, 79.6, 77.2, 57.0, 39.7 ppm; HRMS (ESI): C_26_H_20_Br_2_N_4_ [M+H]^+^: 547.0088; found: 547.0081.

### Characterization of Products 2l

Synthesized according to the general procedure; Mp = 160–161°C (from CH_3_OH); ^1^H NMR (400 MHz, DMSO-d_6_): δ 7.29–7.11 (m, 10H), 6.06 (dd, *J* = 8.4, 0.8 Hz, 2H), 4.58 (dd, *J* = 8.4, 3.6 Hz, 2H), 4.41 (s, 4H), 3.16 (d, *J* = 3.6 Hz, 2H) ppm; ^13^C NMR (100 MHz, DMSO-d_6_) δ 163.2, 160.8, 145.5, 134.2, 134.1, 130.8, 129.9, 129.8, 121.3, 115.8, 115.6, 101.9, 77.4, 55.6, 39.5 ppm; HRMS (ESI): C_26_H_20_F_2_N_4_ [M+H]^+^: 427.1689; found: 427.1687.

### Characterization of Products 2m

Synthesized according to the general procedure; Mp = 163–165°C (from CH_3_OH); ^1^H NMR (500 MHz, CDCl_3_): δ 7.38(d, *J* = 8 Hz, 2H), 7.23 (d, *J* = 2 Hz, 2H), 7.01 (dd, *J* = 9.5 3 Hz, 2H), 6.73 (d, *J* = 1.5 Hz, 2H), 5.94 (dd, *J* = 8, 1 Hz, 2H), 4.79 (dd, *J* = 8.5, 4 Hz, 2H), 4.26 (d, *J* = 3 4H), 3.43 (d, *J* = 3.5 Hz, 2H) ppm; ^13^C NMR (125 MHz, CDCl_3_) δ 144.0, 136.6, 133.0, 132.3, 130.9, 129.9, 128.8, 126.4, 120.3, 103.3, 79.9, 56.5, 39.6 ppm; HRMS (ESI): C_26_H_18_Cl_4_N_4_ [M+H]^+^: 527.0319; found: 527.0312.

### Characterization of Products 2n

Synthesized according to the general procedure; Mp = 157–158°C (from CH_3_OH); ^1^H NMR (500 MHz, CDCl_3_): δ 7.05–7.00 (m, 2H), 6.98–6.89 (m, 4H), 6.74 (d, *J* = 1.5 Hz, 2H), 5.89 (dd, *J* = 8, 1.5 Hz, 2H), 4.75 (q, *J* = 4 Hz, 2H), 4.31 (s, 4H), 3.41 (d, *J* = 4 Hz, 2H) ppm; ^13^C NMR (125 MHz, CDCl_3_) δ 159.6, 157.7, 157.4, 157.3, 155.4, 143.9, 129.8, 125.4, 125.3, 125.2, 125.2, 120.2, 117.0, 116.8, 116.4, 116.3, 116.2, 116.1, 115.7, 115.5, 103.1, 79.9, 51.3, 39.3 ppm; HRMS (ESI): C_26_H_18_F_4_N_4_ [M+H]^+^: 463.1501; found: 463.1493.

### Characterization of Products 2o

Synthesized according to the general procedure; Mp = 133–135°C (from CH_3_OH); ^1^H NMR (500 MHz, CDCl_3_): δ 7.19–7.14(td, *J* = 8.5, 6.5 Hz, 2H), 6.89–6.82 (m, 4H), 6.72 (s, 2H), 5.83 (dd, *J* = 8, 1 Hz, 2H), 4.66 (dd, *J* = 6.4 2.8 Hz, 2H), 4.26 (s, 4H), 3.34 (d, *J* = 3.5 Hz, 2H) ppm; ^13^C NMR (125 MHz, CDCl_3_) δ 164.0, 163.9, 162.0, 161.9, 161.8, 159.9, 159.8, 143.8, 130.6, 130.5, 129.4, 120.5, 119.4, 119.3, 111.8, 111.7, 111.6, 104.6, 104.4, 104.2, 103.0, 79.6, 51.1, 39.59 ppm; HRMS (ESI): C_26_H_18_F_4_N_4_ [M+H]^+^: 463.1501; found: 463.1487.

### Characterization of Products 2p

Synthesized according to the general procedure; Mp = 149–151°C (from CH_3_OH); ^1^H NMR (500 MHz, CDCl_3_): δ 7.12 (dt, *J* = 10 8.5 Hz, 2H), 7.01–6.97 (m, 2H), 6.93–6.90 (m, 2H), 6.72 (d, *J* = 1.5 Hz, 2H), 5.90 (dd, *J* = 8.5 1.2 Hz, 2H), 4.78 (dd, *J* = 8.5, 4 Hz, 2H), 4.27 (d, *J* = 3 4H), 3.43 (d, *J* = 3.5 Hz, 2H) ppm; ^13^C NMR (125 MHz, CDCl_3_) δ 151.5, 151.4, 151.0, 150.9, 149.6, 149.5, 149.0, 148.9, 144.0, 133.4, 133.3, 129.9, 123.2, 120.3, 117.9, 117.8, 116.1, 116.0, 103.4, 79.9, 56.5, 39.7 ppm; HRMS (ESI):C_26_H_18_F_4_N_4_ [M+H]^+^: 463.1501; found: 463.1500.

### Characterization of Products 2q

Synthesized according to the general procedure; Mp = 185–186°C (from CH_3_OH); ^1^H NMR (500 MHz, CDCl_3_): δ 7.52 (dd, *J* = 9, 5.5 Hz, 2H), 6.99 (dd, *J* = 9 3 Hz, 2H), 6.89–6.85 (m, 2H), 5.9 (d, *J* = 1, Hz, 2H), 5.97 (d, *J* = 6 Hz, 2H), 4.88 (dd, *J* = 8.5, 3.5 Hz, 2H), 4.39–4.31 (m, 4H), 4.36 (d, *J* = 3.5 Hz, 2H) ppm; ^13^C NMR (125 MHz, CDCl_3_) δ 163.1, 161.1, 144.1, 137.9, 134.5, 130.2, 120.1, 116.6, 116.5, 116.1, 115.9, 103.3, 80.1, 77.2, 57.4, 39.4 ppm; HRMS (ESI): C_26_H_18_Br_2_F_2_N_4_ [M+H]^+^: 582.9899; found: 582.9886.

### Characterization of Products 2r

Synthesized according to the general procedure; Mp = 132–133°C (from CH_3_OH); ^1^H NMR (500 MHz, CDCl_3_): δ 6.64 (d, *J* = 1.5 Hz, 2H), 5.86 (dd, *J* = 8.5 1.5 Hz, 2H), 4.70 (dd, *J* = 8 3.5 Hz, 2H), 3.34 (d, *J* = 3.5 Hz, 2H), 2.97 (s, 6H) ppm; ^13^C NMR (125 MHz, CDCl_3_) δ 144.7, 130.5, 121.0, 102.9, 78.4, 40.9, 39.6 ppm; HRMS (ESI): C_14_H_14_N_4_ [M+H]^+^: 239.1252; found: 239.1249.

### Characterization of Products 2s

Synthesized according to the general procedure; Mp = 128–129°C (from CH_3_OH); ^1^H NMR (500 MHz, CDCl_3_): δ 6.66 (d, *J* = 1.5 Hz, 2H), 5.89 (dd, *J* = 8 1.5 Hz, 2H), 4.70 (dd, *J* = 8.5 4 Hz, 2H), 3.36 (d, *J* = 3.5 Hz, 2H), 3.12–3.03 (m, 4H), 1.59–1.53 (m, 4H), 0.91 (t, *J* = 7.5 Hz, 6H) ppm; ^13^C NMR (125 MHz, CDCl_3_) δ 144.2, 129.8, 121.2, 102.5, 78.0, 55.9, 39.9, 23.1, 10.8 ppm; HRMS (ESI): C_18_H_22_N_4_ [M+H]^+^: 295.1878; found: 295.1876.

### Characterization of Products 2t

Synthesized according to the general procedure; Mp = 99–101°C (from CH_3_OH); ^1^H NMR (500 MHz, CDCl_3_): δ 6.75 (d, *J* = 1.5 Hz, 2H), 5.97 (d, *J* = 8 Hz, 2H), 4.73–4.70 (m, 2H), 3.36–3.35 (m, 2H), 3.06–2.95 (m, 4H), 1.00–0.93(m, 2H), 0.61–0.58 (m, 4H), 0.21 (q, *J* = 5 Hz, 4H) ppm; ^13^C NMR (125 MHz, CDCl_3_) δ 143.9, 130.0, 121.3, 102.4, 78.2, 77.3, 77.1, 76.9, 58.4, 39.9, 10.8, 3.4 ppm; HRMS (ESI):C_18_H_22_N_4_ [M+H]^+^: 319.1878; found: 319.1869.

### Characterization of Products 3a

Synthesized according to the general procedure; ^1^H NMR (400 MHz, DMSO-d_6_): δ 7.47 (dd, *J* = 7.6 1.6 Hz, 1H), 7.39–7.28 (m, 3H), 7.08 (s, 1H), 6.14 (d, *J* = 5.2 Hz 1H), 4.65–4.61 (m, 2H), 4.43 (d, *J* = 16 Hz 1H), 2.32–2.23 (m, 1H), 2.05–1.99 (m, 1H) 1.85–1.79 (m, 1H), 1.59–1.50 (m, 1H) ppm; ^13^C NMR (100 MHz, DMSO-d_6_) δ 146.9, 135.8, 132.8, 130.0, 129.8, 129.6, 127.9, 123.0, 75.5, 74.4, 53.1, 28.0, 17.2 ppm; HRMS (ESI): C_13_H_14_N_2_O [M+H]^+^: 214.1187; found: 214.1191.

## Results and Discussion

The majority of substituents of the photochemical reaction substrate, 1,4-dihydropyridine, at the −3-position are ester groups, with few reports of attachment of other groups at this position (Jin et al., 1998; Hilgeroth and Heinemann, 1999; Hilgeroth et al., 1999).

We initially used commercially available -cyano substituted pyridine as the model substrate under irradiation with blue LED light (Table 1).

**Table 1:** Optimization of the reaction parameters^a^.

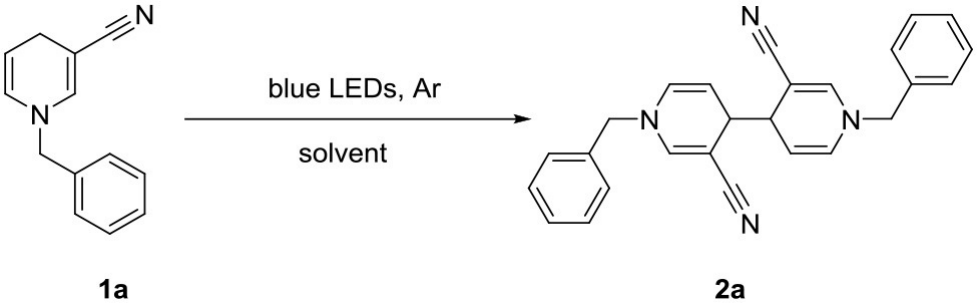					
**Entry**	**Solvent**	**Atmosphere**	**Light source**^**d**^	**Time (h)**^**b**^	**Yield (%)**^**c**^
1	Ace	Ar	LED (410 nm)	59	54
2	THF	Ar	LED (410 nm)	57	46
3	MeOH	Ar	LED (410 nm)	67	68
4	95% EtOH	Ar	LED (410 nm)	62	65
5	EtOH/H_2_O (1:1)	Ar	LED (410 nm)	42	33
6	EtOH/H_2_O (1:2)	Ar	LED (410 nm)	39	25
7	EtOH	Ar	LED (410 nm)	72	84
8	EtOH	Air	LED (410 nm)	68	79
9	EtOH	Ar	–	72	Trace
10	EtOH	Ar	LED (365 nm)	82	64
11	EtOH	Ar	LED (450 nm)	78	60
12	EtOH	Ar	Hg-500 W	6	—

a Irradiation of 1a (5 mmol) in various solvents (25 mL) with blue LED lamps at room temperature.

b Reaction time was determined based on complete consumption of 1a.

c Isolated yield.

d *10 W blue LED, high-pressure mercury lamp (500 W)*.

The yield of 2a in acetone (Ace), tetrahydrofuran (THF), methanol, or 95% ethanol (EtOH) was moderate and reaction time was 57–67 h (entries 1–4). Interestingly, the amount of water in solvent was associated with reaction time. To further explore this result, a significant amount of water was added to alcohol. While the reaction time decrease with increasing moisture, yield of the corresponding product 2a was also decreases (entries 5, 6). Next, we examined the effect of an anhydrous solvent on the reaction. As expected, yield of 2a was successfully increased to 84% by limiting the amount of added water (entry 7). Notably, moisture in the air and impurities were contributory factors to decreased yield (entry 8). Data obtained from control experiments confirmed the necessity of light (entry 9). Irradiation with 365 nm and 450 nm sources not only prolonged the reaction time but also reduced yield of 2a (entries 10, 11). Simultaneously, a higher-power high-pressure mercury lamp was used for irradiation. In this case, reaction time was greatly reduced but no product 2a was generated (entry 12).

Irradiation of 1a (5 mmol) in anhydrous EtOH at ambient temperature under Ar atmosphere for 72 h were determined as the optional conditions. Next, we investigated the scope and generality of the photocatalytic coupling reaction 1,4-dihydropyridines by introducing different substituents under the established optimal conditions results are summarized in Table 2.

**Table 2:**
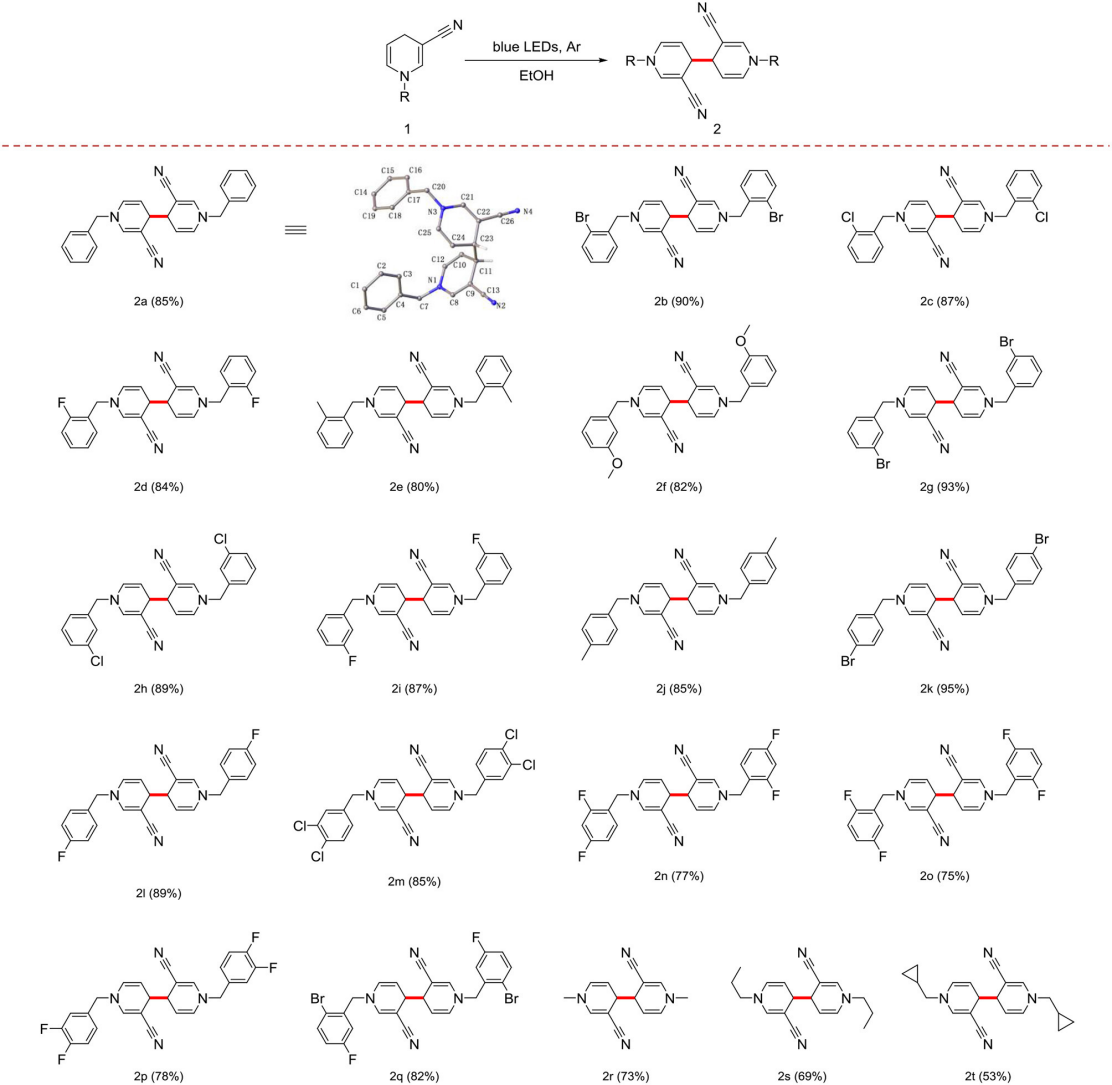
Scope and generality of coupling reactions of 1,4-dihydropyridine derivatives under photocatalytic conditions^a^.

a *Irradiation of 1a (5 mmol) in anhydrous EtOH (25 mL) with blue LED lamps at room temperature. Products were purified via column chromatography using silica gel (200–300 mesh) and percentage yields of the isolated products are presented*.

First, in the absence of a substituent on the benzyl group, yield of the product 2a reached 85%. When the benzyl group contained -F, -Cl, -Br, or -CH_3_ in the ortho-position, yield with electron-withdrawing groups was superior to that with electron-donating groups (Table 2, 2b−2e). In cases where the substituent was in the meta- or para-position, good yield of the target product was obtained (Table 2, 2f−2l). With different positions of the same substituent product yield with the substituent in the para-position was higher than that the ortho- and meta- positions owing to a steric hindrance effect (Table 2, 2d, 2i, and 2l).

Notably, the benzyl group with multiple substituents generated a moderate yield (Table 2, 2m−2q). In addition, the benzyl group at the 1-position could be effectively replaced with an alkyl or cycloalkyl group (Table 2, 2r−2t).

**Figure 2:**
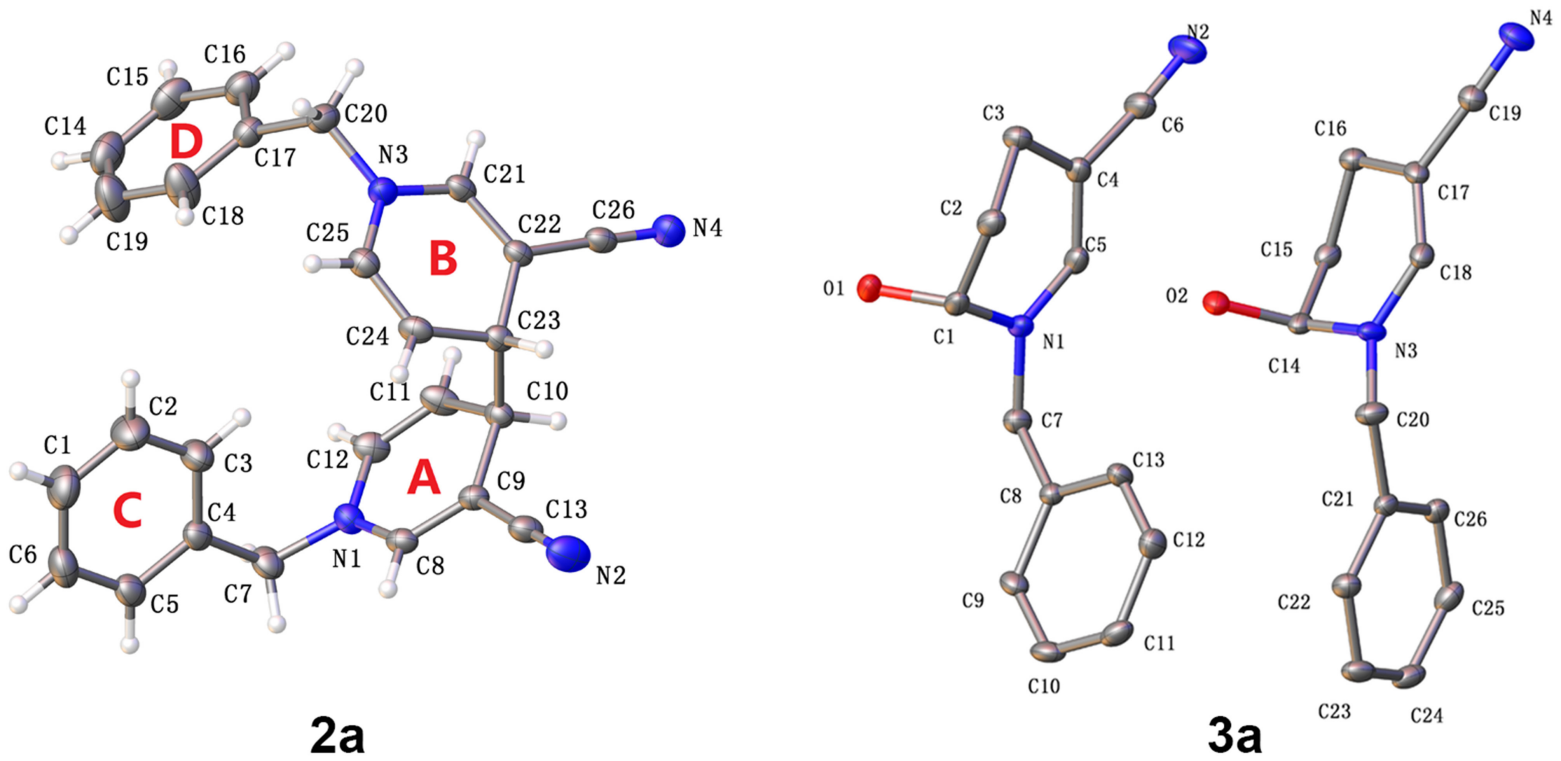
Olex2 diagram (30%) of compound 2a and 3a.

**Scheme 2:**
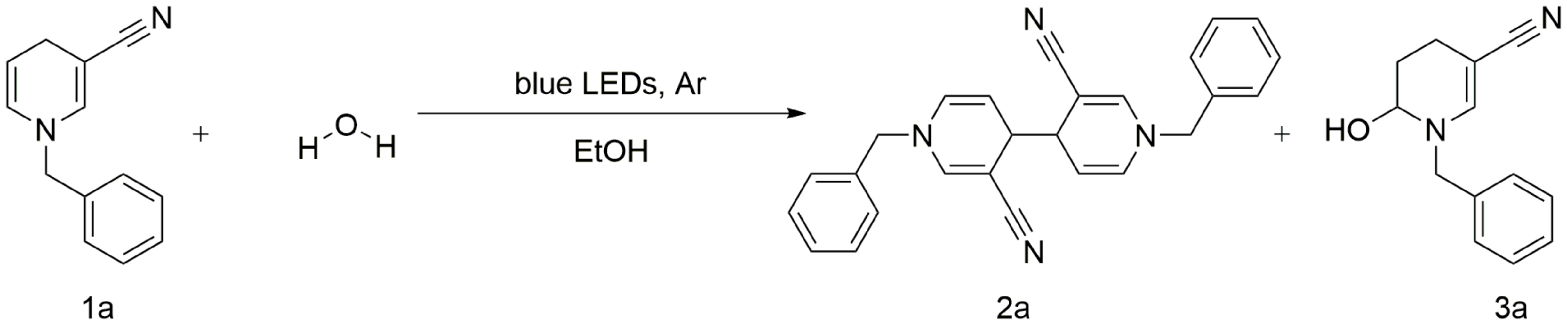
Reaction of 1,4-dihydropyridine derivatives in the presence of water molecules.

The molecular structures of 2a and 3a are depicted in Figure 2 (CCDC 1876160^1^ and 1497344^2^). In the case of 4,4′-linked dimers, a maximum of three diastereoisomers could exist with, possible RR, SS or RS configurations. The reaction generated a levoisomer, which was confirmed via single crystal X-ray crystallography (Figure 2). In 2a, the two molecules were not on different sides as envisaged. Bipyridine compounds mainly consisted of two pyridine rings [A (N1-C8-C12), B (N3-C21-C25)] and two benzene rings[C (C1-C6), D (C14-C19)]. No molecular hydrogen bonds were observed. Unexpectedly, in contrast to previous olefin and water reactions requiring high temperature or high pressure under acidic conditions, photocatalysis promoted reaction of water molecules with the double bond to form the by-product 3a (Scheme 2). Moreover, π-π stacking was not evident in the X-ray structures of 2a and 3a.

**Scheme 3:**
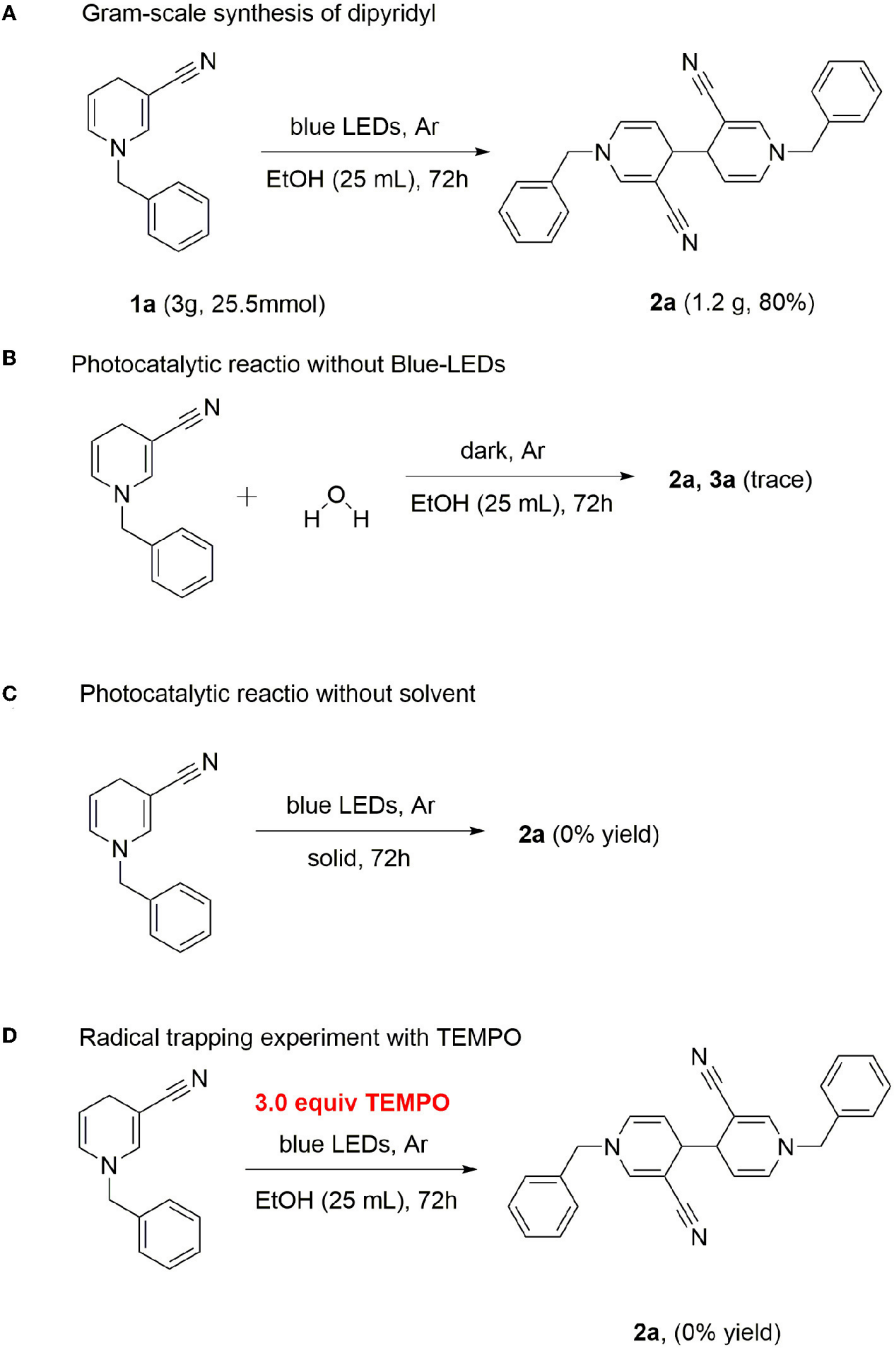
Gram-scale synthesis and control experiments.

Gram-scale synthesis of dipyridyl was additionally conducted. In general, photoreaction is difficult at high concentrations. However, dipyridyl 2a was obtained with a good yield, even at a scale of 25.5 mmol (80%) (Scheme 3A), supporting the practical application of this method in the industrial field.

Water was added in the case of a standard reaction. The photocatalytic reaction revealed that the reaction was completely inhibited in the absence of light, indicating that continuous irradiation of visible light is essential for this photo-catalytic conversion (Scheme 3B). Next, we used the solid-phase illumination method for analysis. However, 2a was not produced in the system, indicating the importance of the solvent (Scheme 3C). To gain insights into the underlying mechanism, control experiments were performed. Since several photocatalytic coupling reactions proceed via the free radical pathway, we performed our model reaction in the presence of the radical scavenger 2,2,6,6-tetramethyl-1-piperidinyloxy (TEMPO), under optimized reaction conditions. In the presence of TEMPO as the scavenger, no product 2a was detected (Scheme 3D).

**Figure 3:**
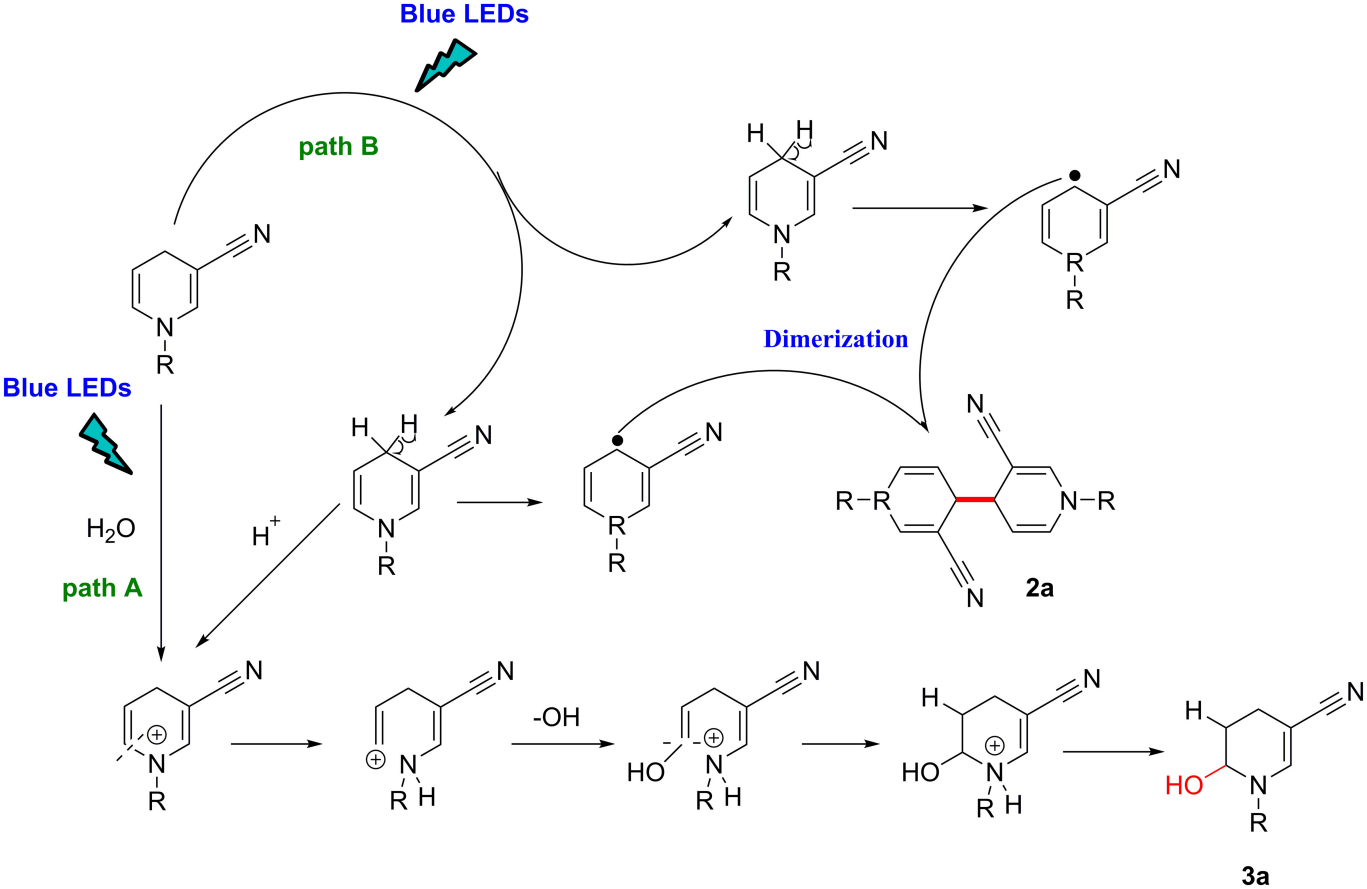
Proposed mechanism.

Based on our findings and previous reports (Deb et al., 2017; Rahaman et al., 2018), an outline of the potential mechanism is illustrated in Figure 3.

Under blue-LED light irradiation, if the reaction system contains water molecules, paths A and B are simultaneously activated, the methylene in the para position is activated to single electron methyl radical, while the proton attacks the π-electron cloud on the double bond, and generates a positively charged intermediate on the double bond carbon through the transition state, afterward the hydroxyl radical attacks the positive ion to form the product 3a (path A). By contrast, in the absence of moisture, the methylene group is homogenized by light. The single-electron methylene group is highly reactive with a strong tendency to pair electrons and dimerizes with another single electron methylene collision to forms a new C–C bond (path B).

## Conclusion

In summary, we have developed a C–C coupling method for pyridine compounds via photocatalysis, which represents a novel, efficient and green approach for selective C–C coupling under mild reaction conditions. Notably, no catalyst or precious metal is required for completion of the reaction and anhydrous ethanol is used as the solvent. The reaction step is relatively simple, which makes construction of the C–C bond more sustainable. Further investigations into the applicability of this methodology for other organic reactions are currently underway. The photo-mediated C–C coupling reaction described in this study should aid in the design of more interesting, useful, and sustainable reactions in the future.

## Data Availability Statement

All datasets generated for this study are included in the article/Supplementary Material.

## Author Contributions

SC synthesized all of the compounds with the help of HZ, CL, PZ, and WS. QZ supervised this work and wrote the paper with the help of SC.

### Conflict of Interest

The authors declare that the research was conducted in the absence of any commercial or financial relationships that could be construed as a potential conflict of interest.

## Footnotes

^1^Crystallographic data for the compound 2a has been deposited with the CCDC 1876160.

^2^Crystallographic data for the compound 3a has been deposited with the CCDC 1497344.
