# Supplementary material for: Visible-Light-Induced C–C Coupling Reaction to Synthesize Bipyridine From 3-Cyano-1,4-Dihydropyridines
Source: Front Chem. 2020 Jan 17;7:940. doi: 10.3389/fchem.2019.00940 (PMC6978658; doi:10.3389/fchem.2019.00940)

**Electronic Supplementary Information**

**Visible-light-induced C-C coupling reaction to synthesize bipyridine from 3-cyano-1,4-dihydropyridines**

Shijun Chen,^[a]^ Qidi Zhong ,*^[a]^ Hao Zhu,*^[b]^ Chunyan Liu,^[a]^ Pengyu Zhuang,^[a]^ and Wuji Sun,^[b]^

*a* *School of Pharmacy, North China University of Science and Technology , Tangshan, Hebei, 063210, P. R. China.*

*b* *School of Public Health, North China University of Science and Technology, Tangshan, Hebei, 063210, P. R. China.*

*E-mail:* [*[Qidizhong@hotmail.com](mailto:nick8110@163.com)*](mailto:Qidizhong@hotmail.com)*[,](mailto:nick8110@163.com)* [*haoyue570126@163.com*](mailto:haoyue570126@163.com)

# Table of the contents

X-ray crystallographic data for **2a and 3a** ……………………………………………………S2-S3

1H NMR and 13C NMR spectra of **2a-2t**… S4

# Molecular structure and crystallographic data of 2a

**
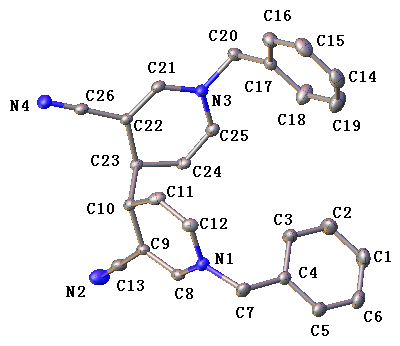
**

Figure S1. X-ray crystal structure of 2a

**Table S1**. Crystal data and structure refinement for **2a**

Formula C_26_H_22_N_4_

CCDC number 1876160

Formula weight 390.47

Temperature (K) 296（2）

Wavelength (Å) 0.71073

Crystal system Monoclinic

Space group P 21/c

Unit cell dimensions a = 11.472(2) Å b= 6.0805(12)Å c = 29.787(6) Å

Volume 2075.9(7) Å^3^

Z 4

Density (calculated) 1.249 Mg/m^3^

Absorption coefficient 0.075 mm^-1^

F (000) 824

Crystal size 0.250 x 0.220 x 0.200 mm^3^

Theta range for data collection 1.368 to 25.000°.

Index ranges -13<=h<=13, -6<=k<=7, -35<=l<=32

Reflections collected 11350

Independent reflections 3661 [R(int) = 0.0307]

Completeness to theta = 25.000° 100 %

Absorption correction Semi-empirical from equivalents

Max. and min. transmission 0.985 and 0.981

Refinement method Full-matrix least-squares on F^2^

Data / restraints / parameters 3661 / 0 / 271

Goodness-of-fit on F^2^ 1.093

Final R indices [I>2sigma(I)] R_1_ =0.0427, wR_2_ = 0.0843

R indices (all data) R_1_ = 0.0563, wR_2_ = 0.0882

Extinction coefficient n/a

Largest diff. peak and hole 0.147 and -0.273 e.Å^-3^

# Molecular structure and crystallographic data of 3a


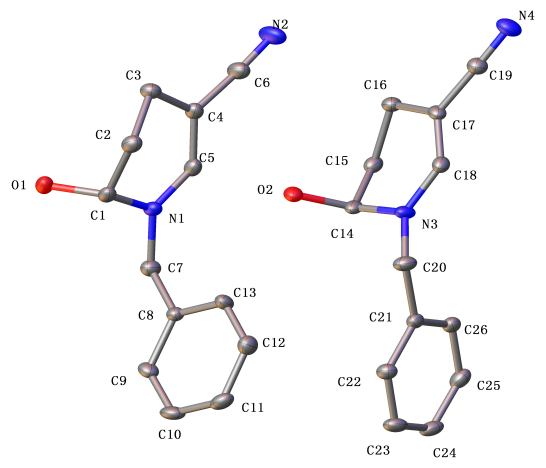

Figure S2. X-ray crystal structure of 3a

**Table S2**. Crystal data and structure refinement for **3a**

Formula C_13_H_14_N_2_O

CCDC number 1497344

Formula weight 214.26

Temperature (K) 293（2）

Wavelength (Å) 0.71073

Crystal system Prism

Space group P 21/c

Unit cell dimensions a=9.933(2) Å

b=22.12(06) Å

c=10.487(3) Å

Volume 2304.2(10) Å^3^

Z 8

Density (calculated) 1.235Mg/m^3^

Absorption coefficient 0.080mm^-1^

F (000) 912

Crystal size 0.220 x 0.160 x 0.140 mm^3^

Theta range for data collection 3.370 to 27.610°.

Index ranges -12<=h<=12, -28<=k<=28, -13<=l<=0

Reflections collected 10566

Independent reflections 5314 [R(int) =0.0447]

Completeness to theta = 25.000° 99.3 %

Absorption correction Semi-empirical from equivalents

Max. and min. transmission 0.9889 and 0.9827

Refinement method Full-matrix least-squares on F^2^

Data / restraints / parameters 5314/ 0 / 292

Goodness-of-fit on F^2^ 1.048

Final R indices [I>2sigma(I)] R_1_ =0.0452, wR_2_ = 0.0783

R indices (all data) R_1_ = 0.0546, wR_2_ =0.0808

Extinction coefficient n/a

Largest diff. peak and hole 0.208 and -0.219 e.Å^-3^

**^1^H-NMR of 2a (400 MHz)**

**
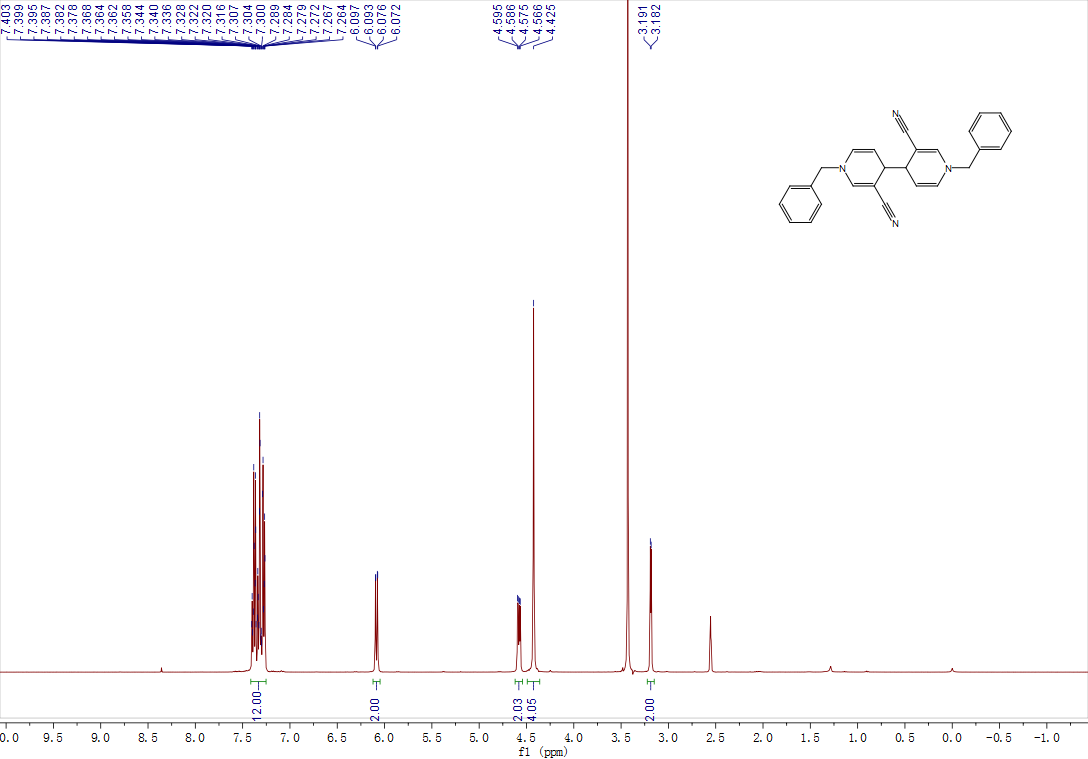
**

**^13^C-NMR of 2a (****100 MHz)**

**
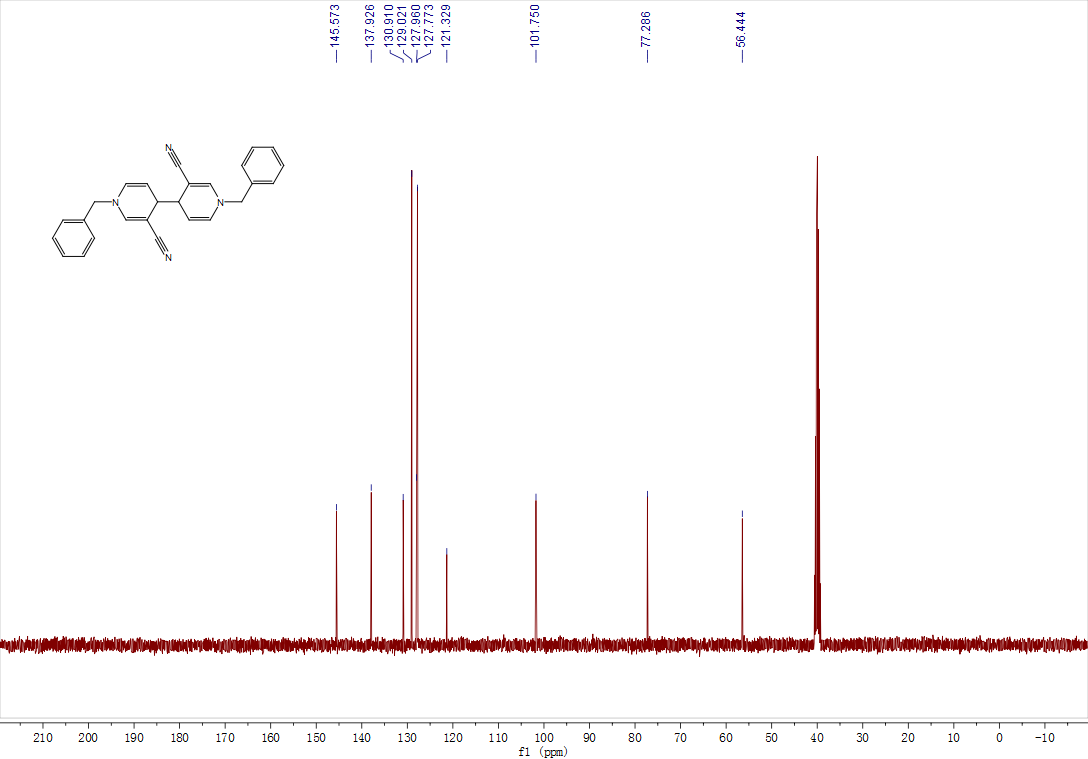
**

**^1^H-NMR of 2b (500 MHz)**

**
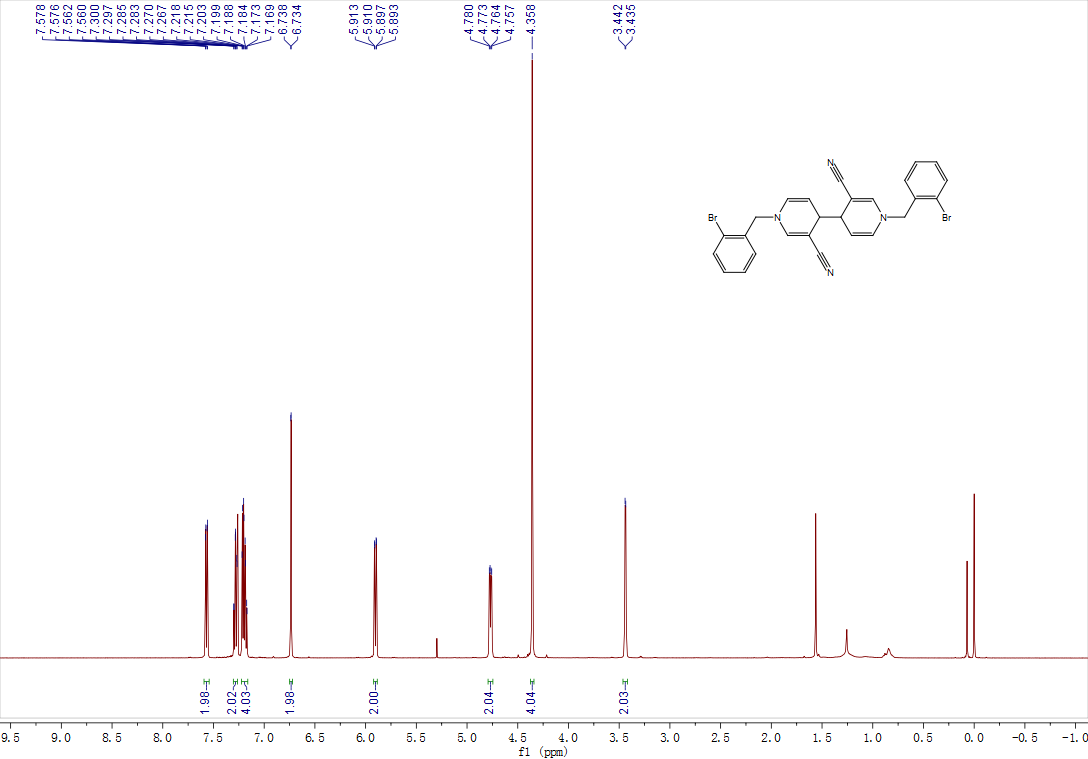
**

**^13^C-NMR of 2b (125 MHz**)


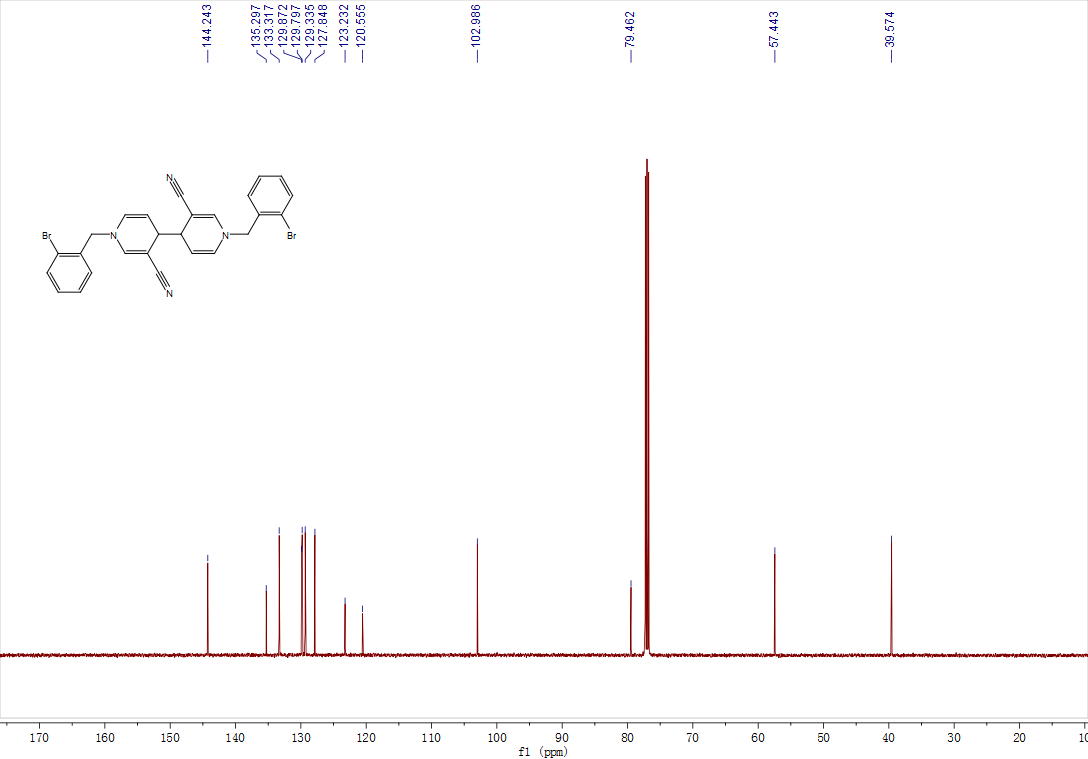


**^1^H-NMR of 2c (400 MHz)**


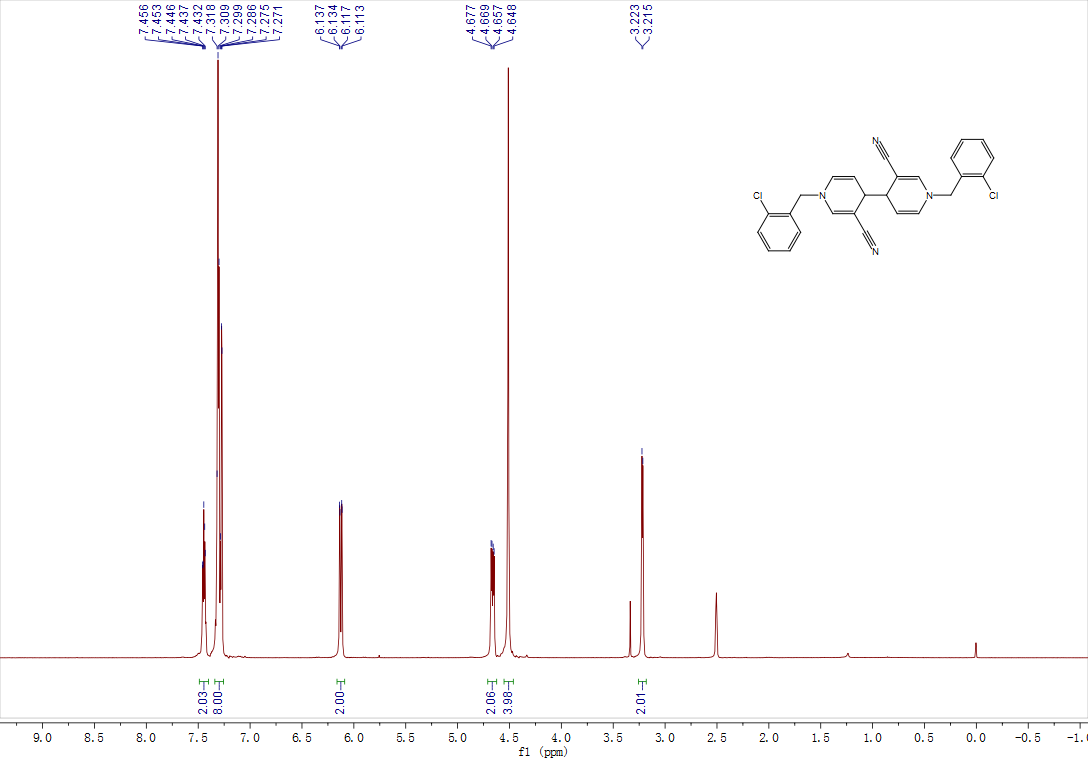


**^13^C-NMR of 2c (100 MHz**)


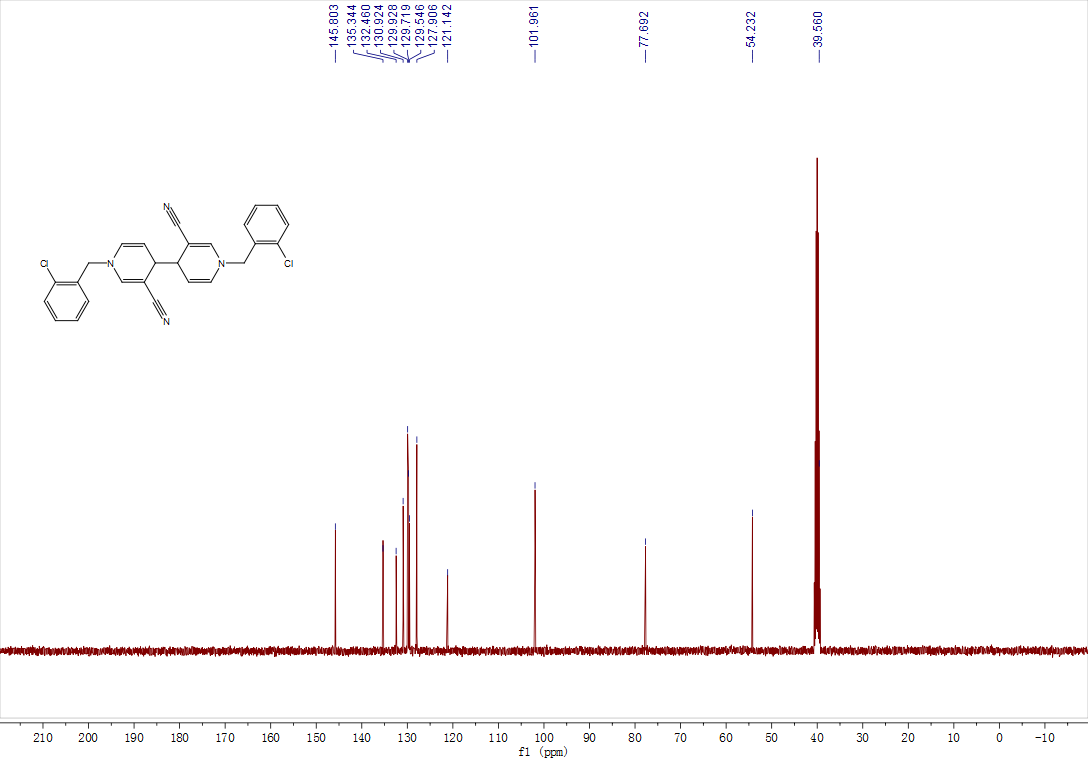


**^1^H-NMR of 2d (400 MHz)**


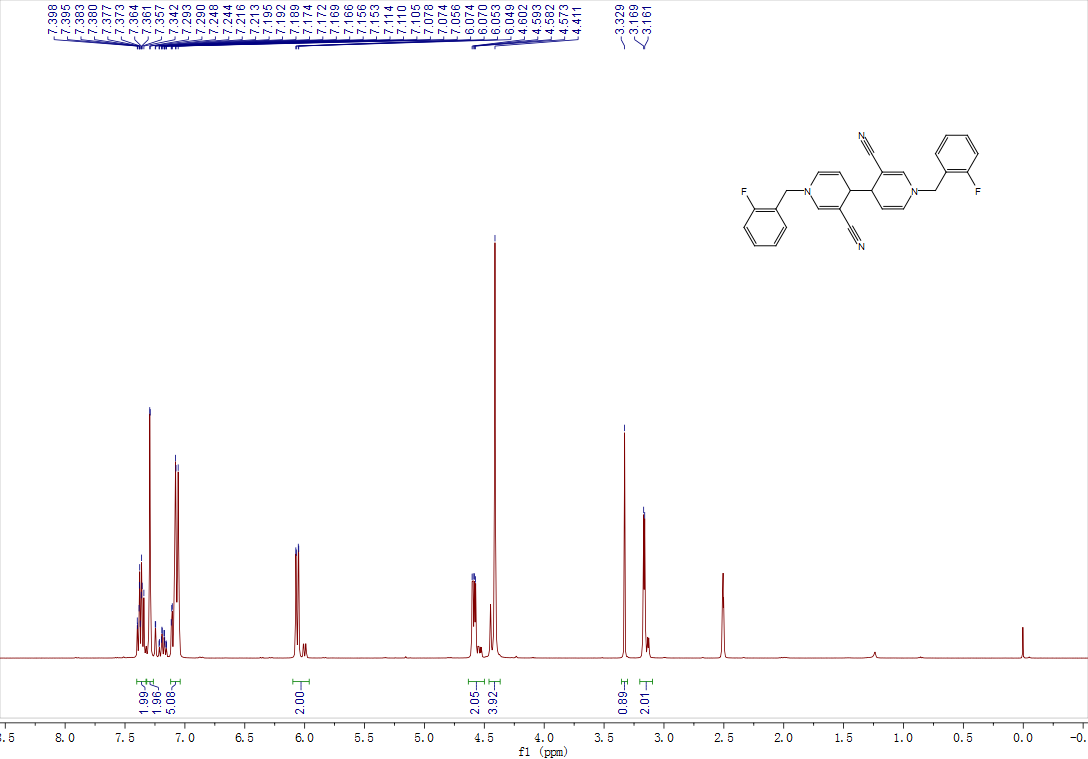


**^13^C-NMR of 2d (100 MHz**)


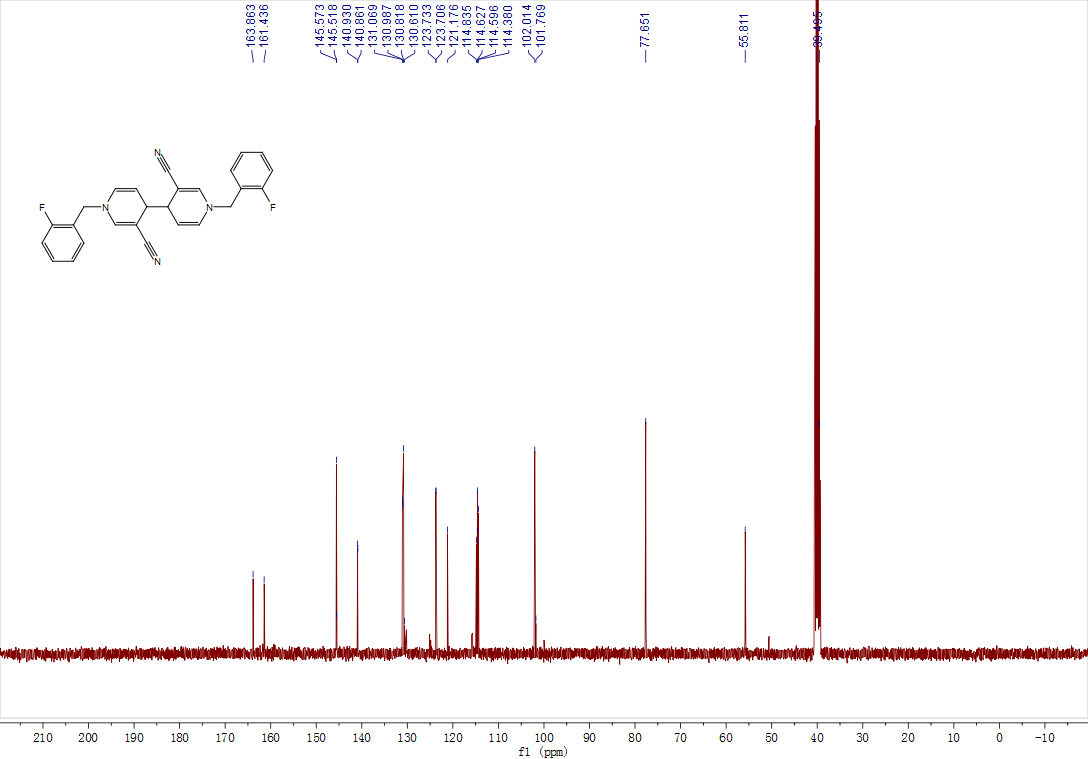


**^1^H-NMR of 2e (400 MHz**)


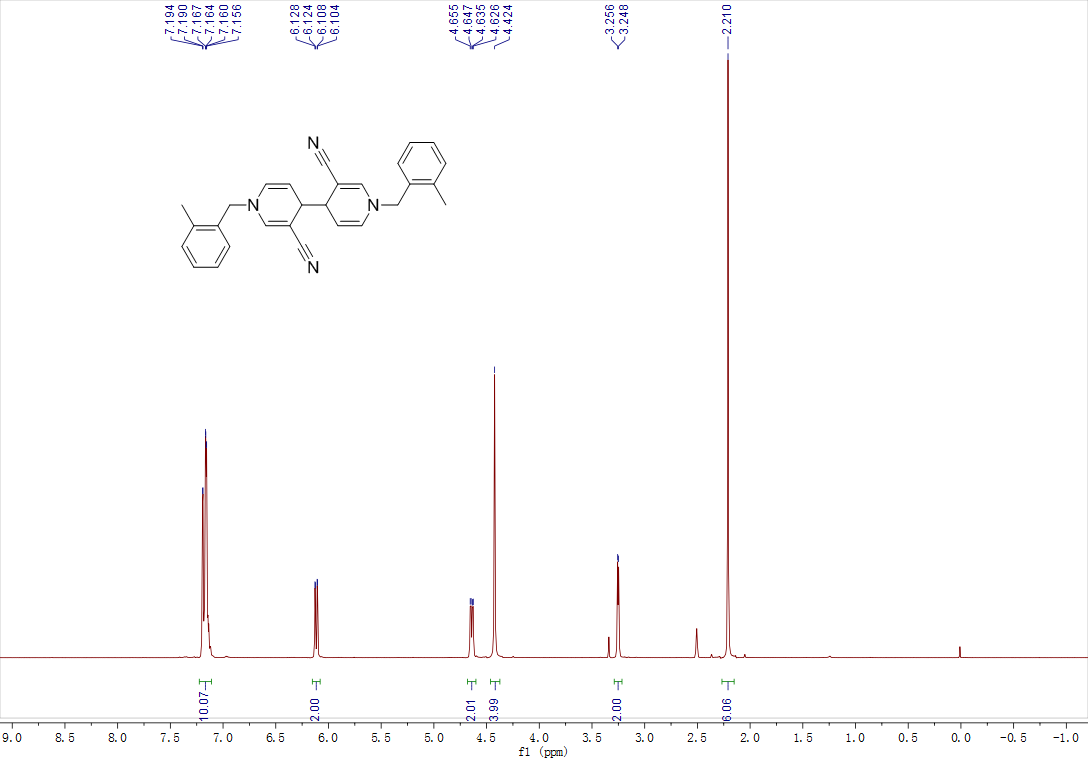


**^13^C-NMR of 2e (100 MHz**)


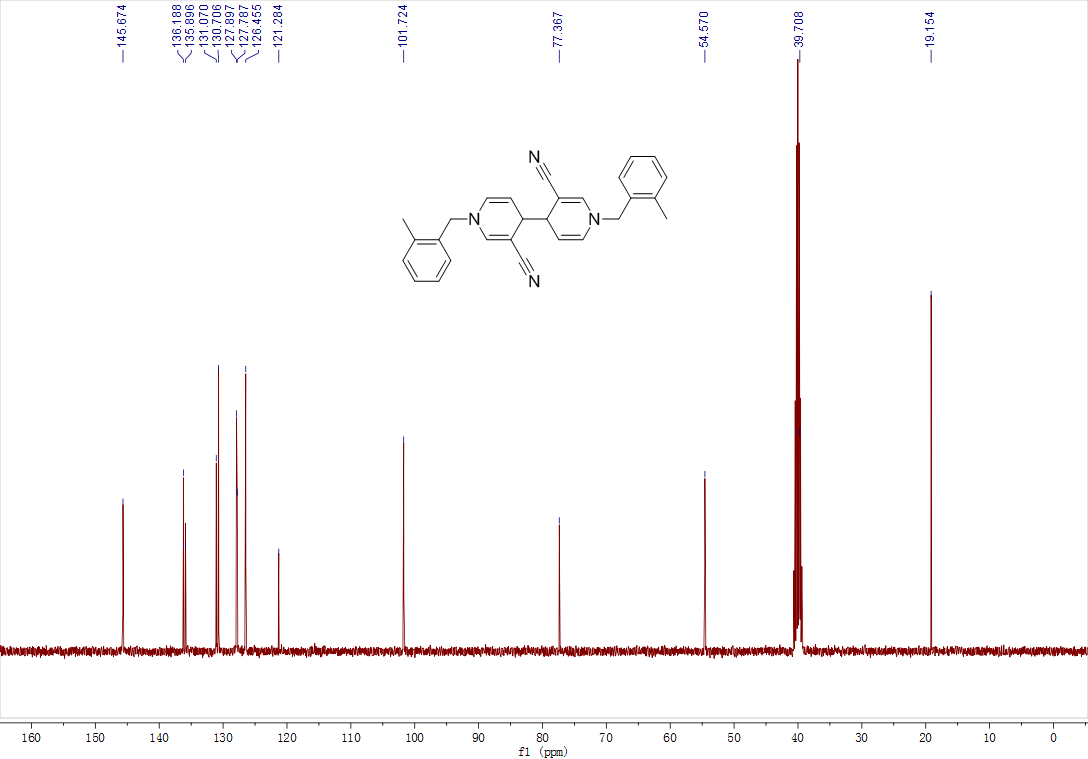


**^1^H-NMR of 2f (500 MHz**)


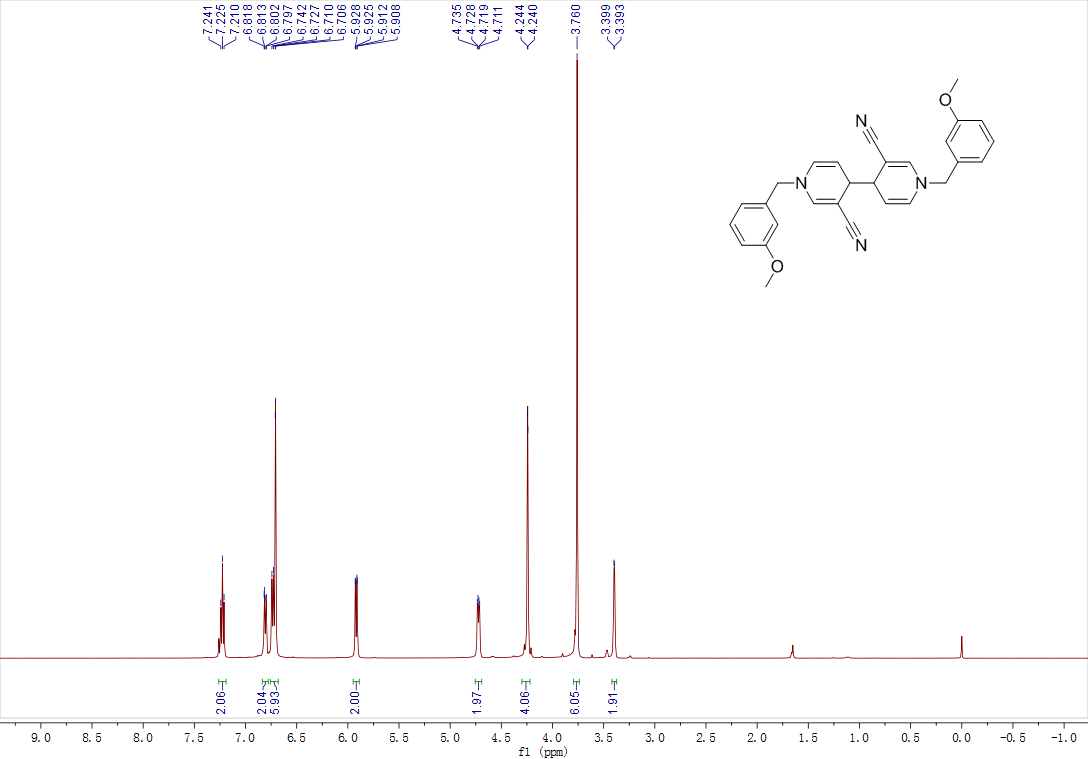


**^13^C-NMR of 2f (125 MHz**)


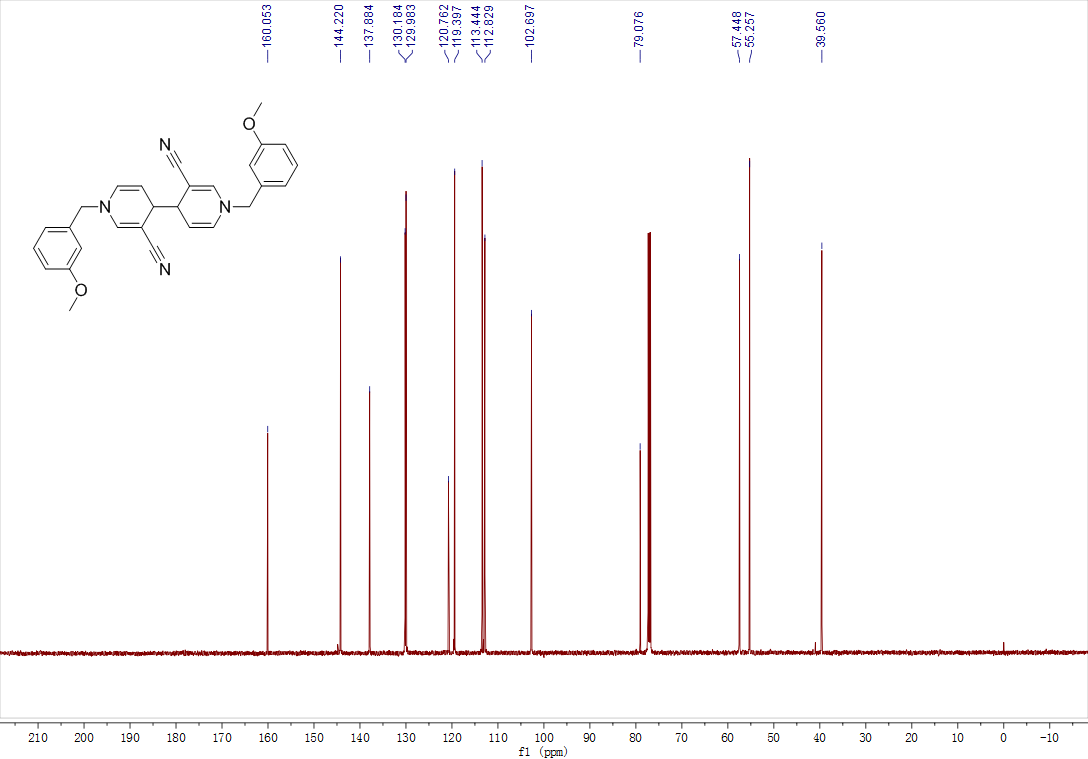


**^1^H-NMR of 2g (500 MHz**)


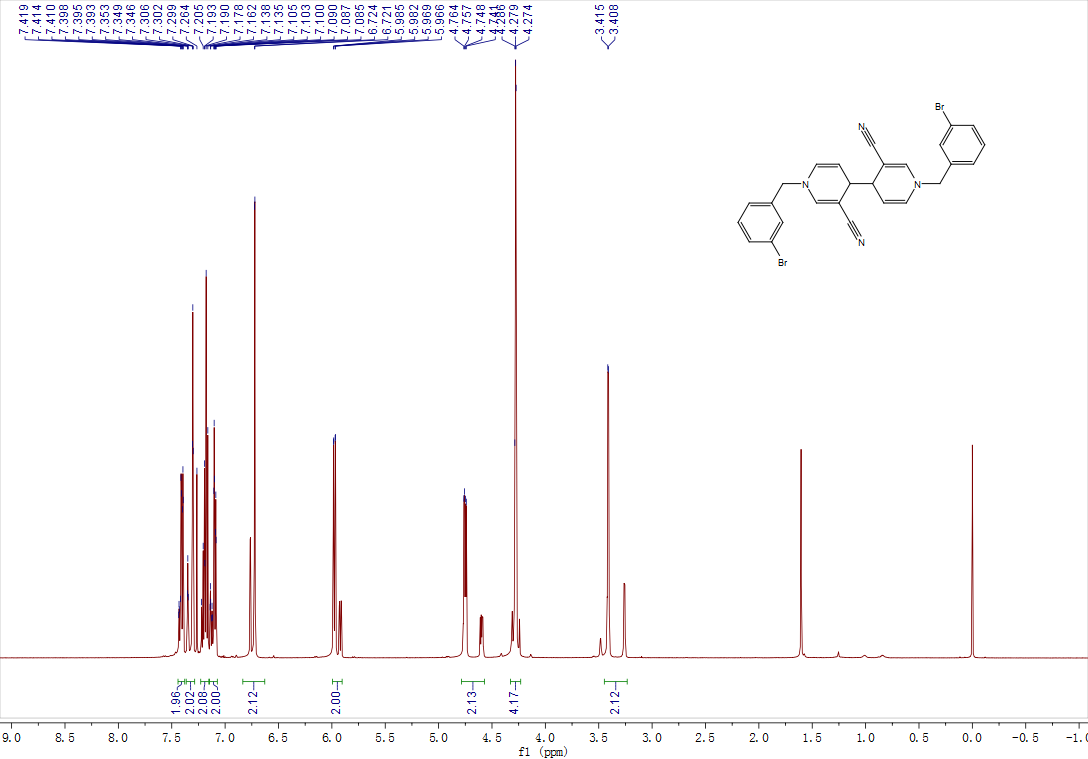


**^13^C-NMR of 2g (125 MHz**)


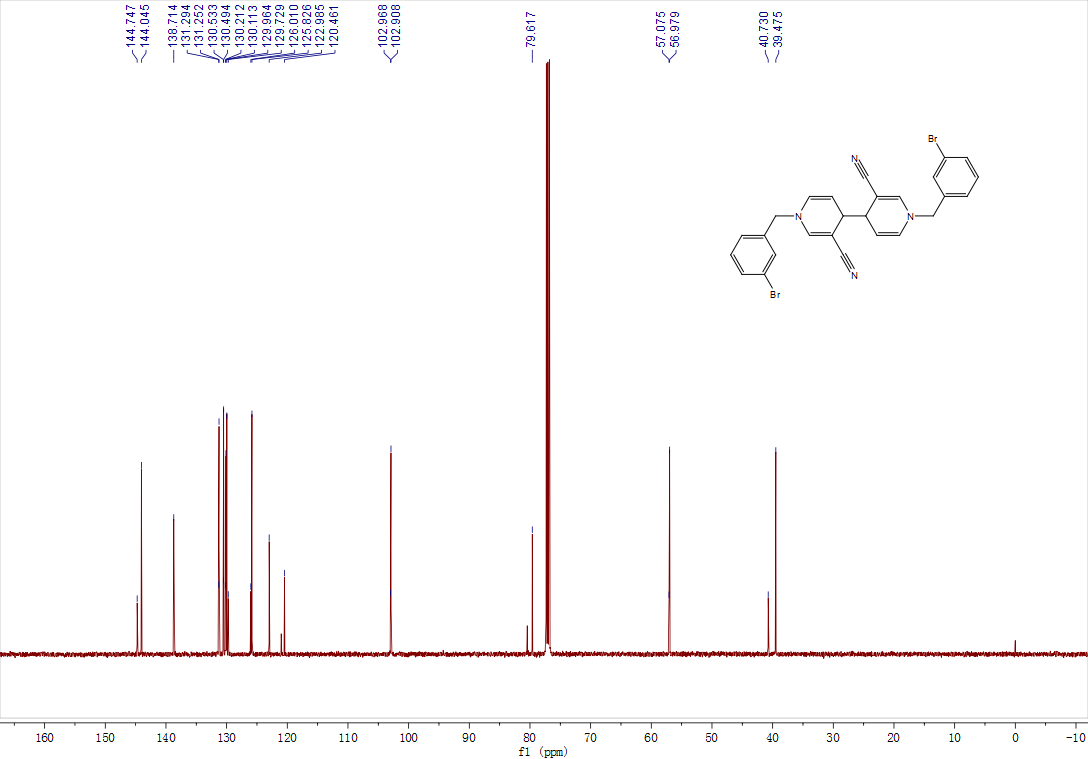


**^1^H-NMR of 2h (500 MHz**)


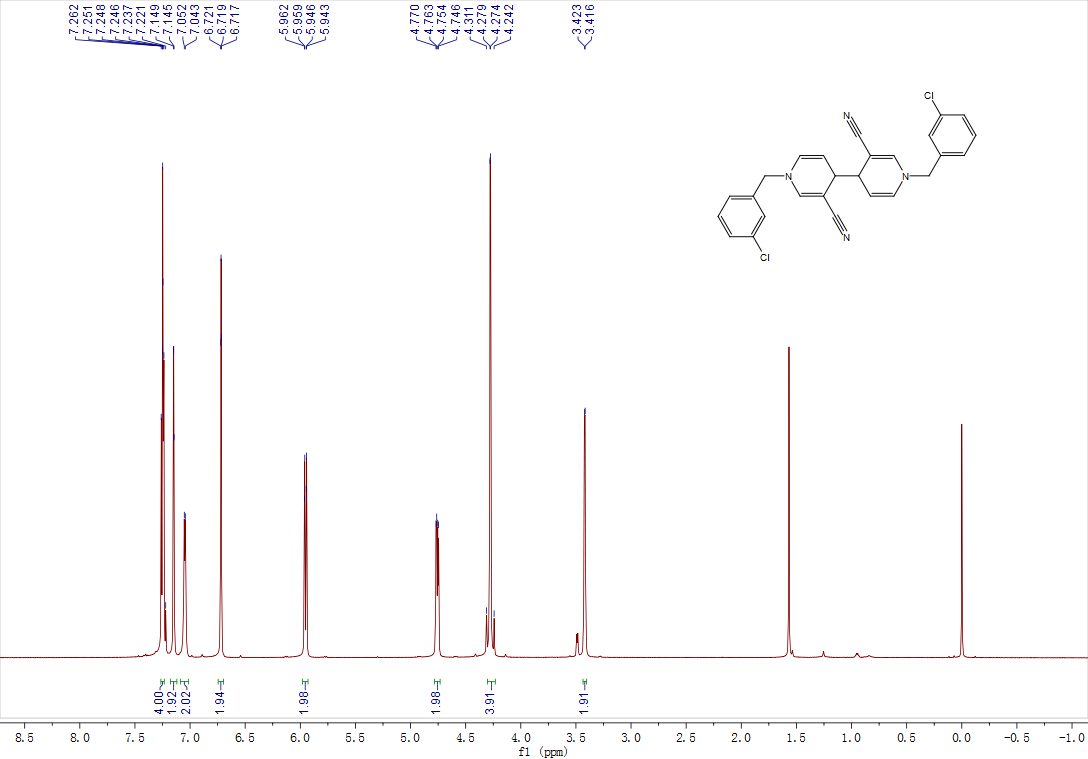


**^13^ C-NMR of 2h (125 MHz**)


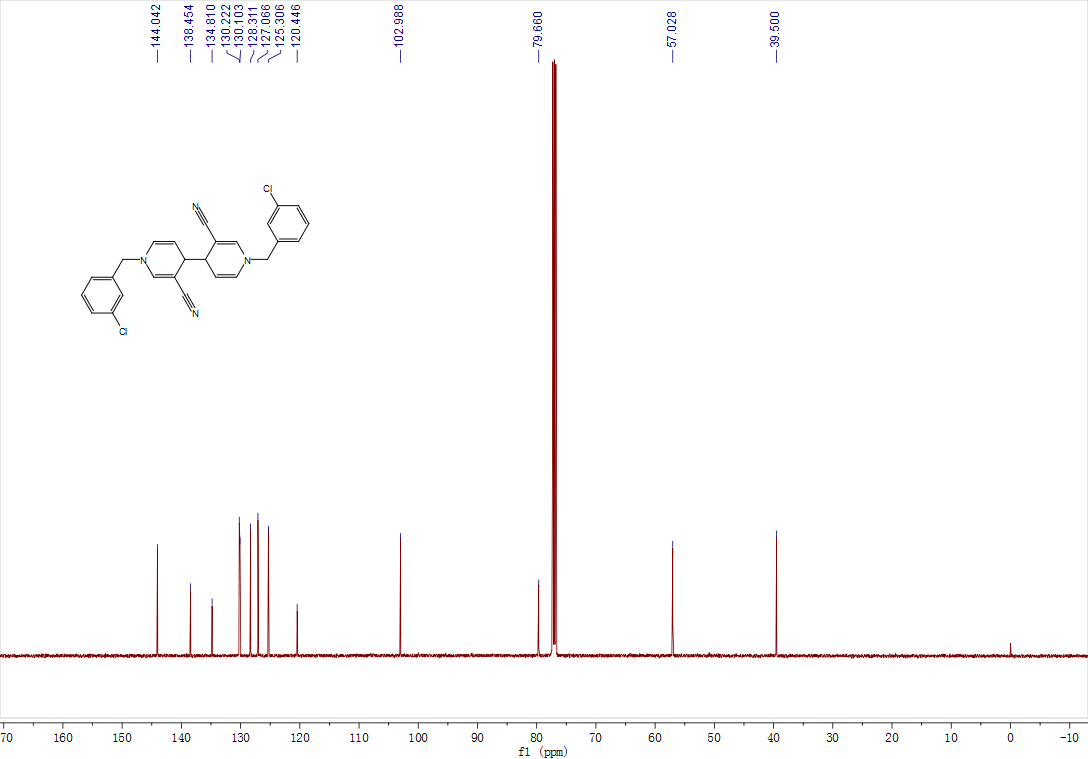


**^1^H-NMR of 2i (500 MHz**)


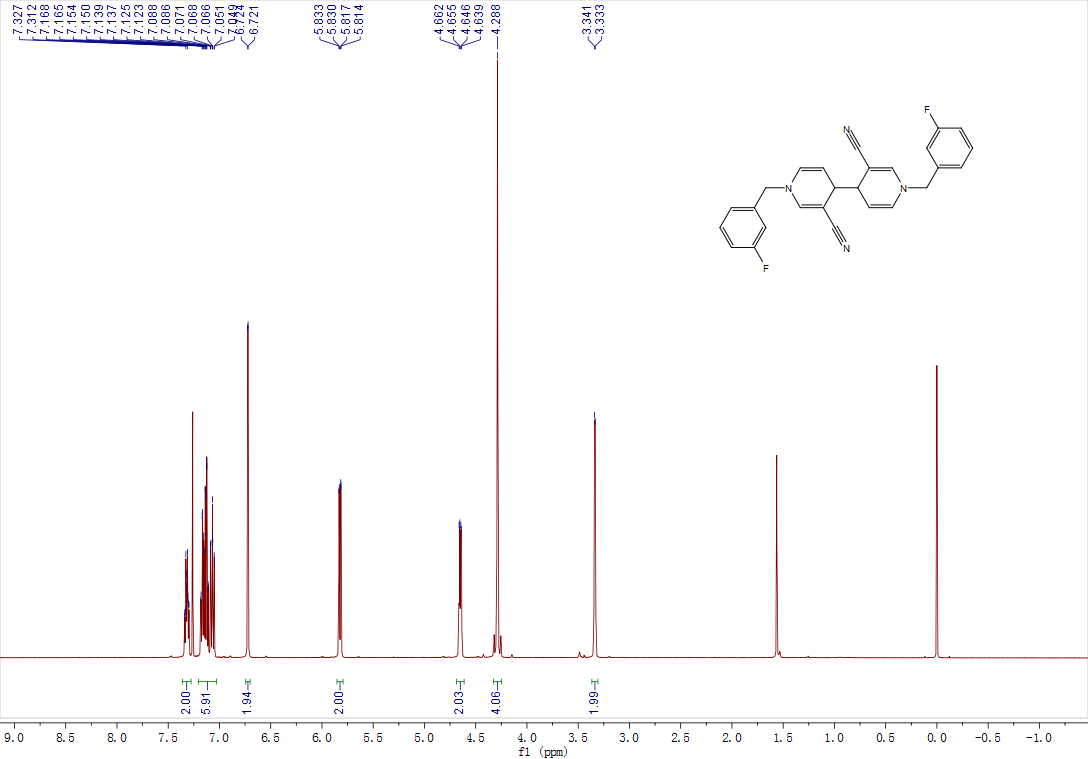


**^13^C-NMR of 2i (125 MHz**)


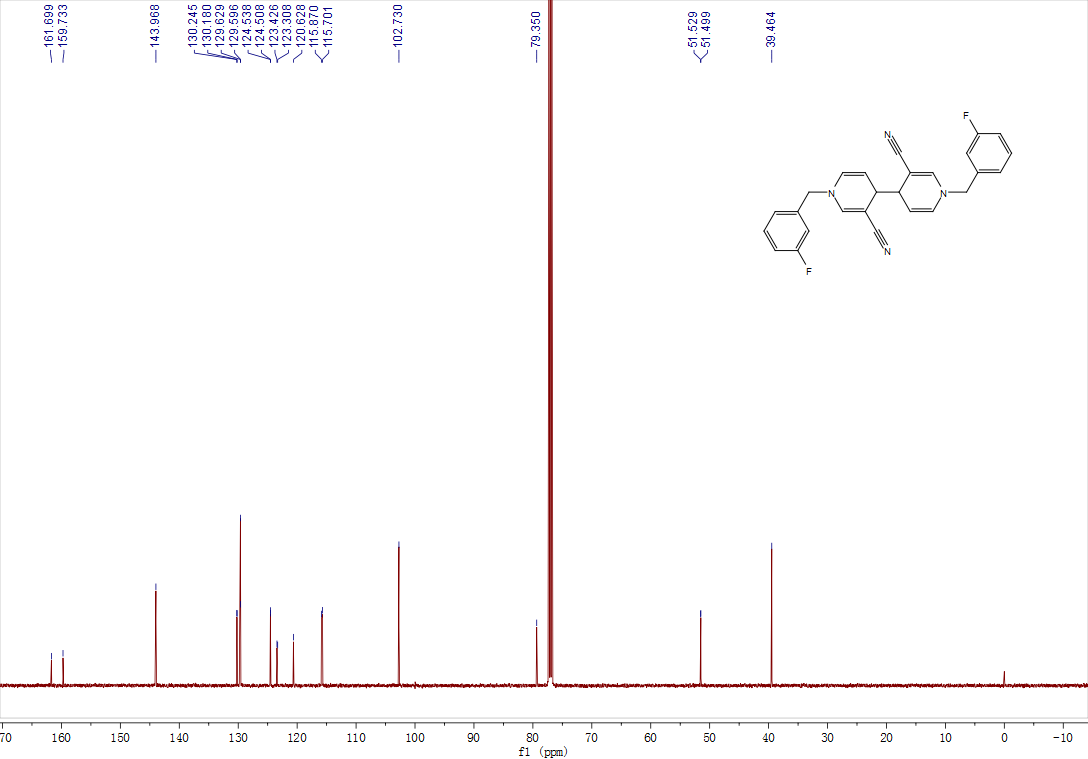


**^1^H-NMR of 2j (400 MHz**)


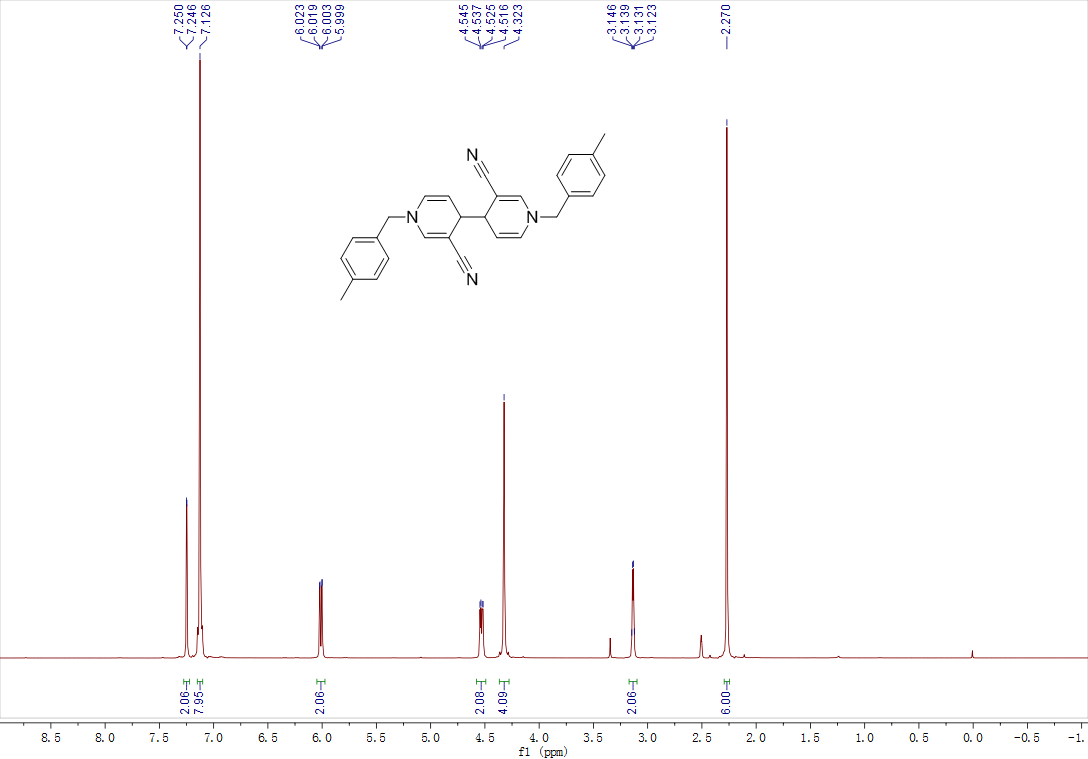


**^13^C-NMR of 2j (100 MHz**)


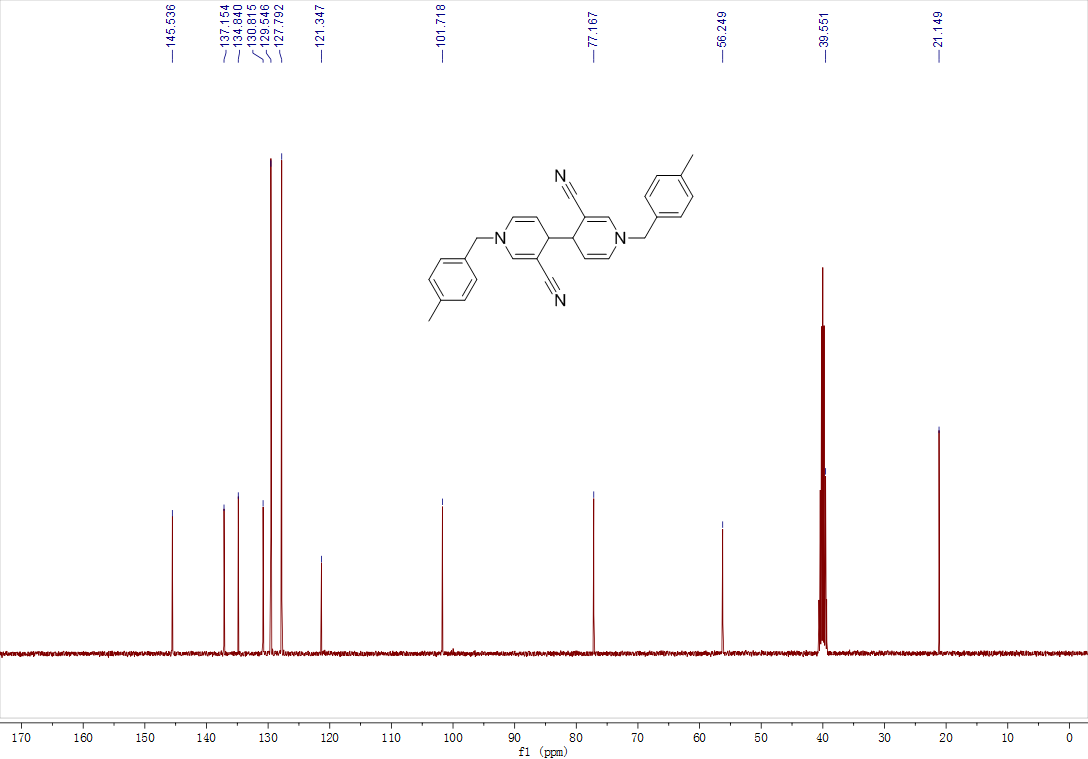


**^13^H-NMR of 2k (500 MHz**)


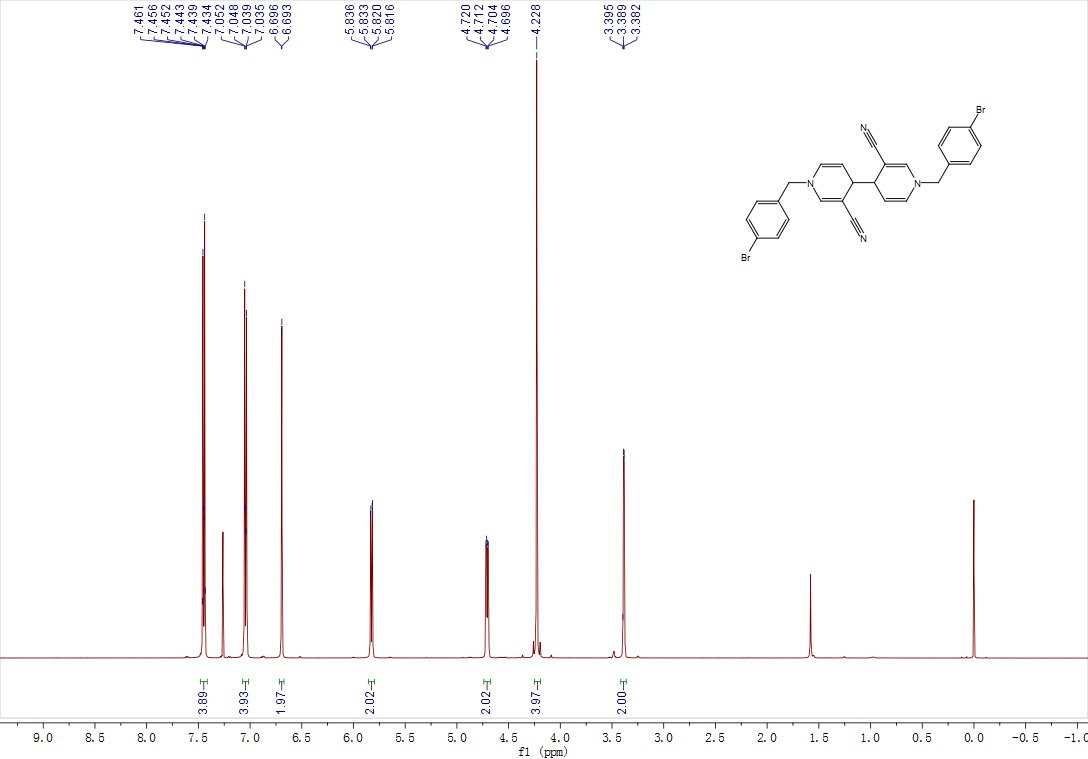


**^13^C-NMR of 2k (125 MHz**)


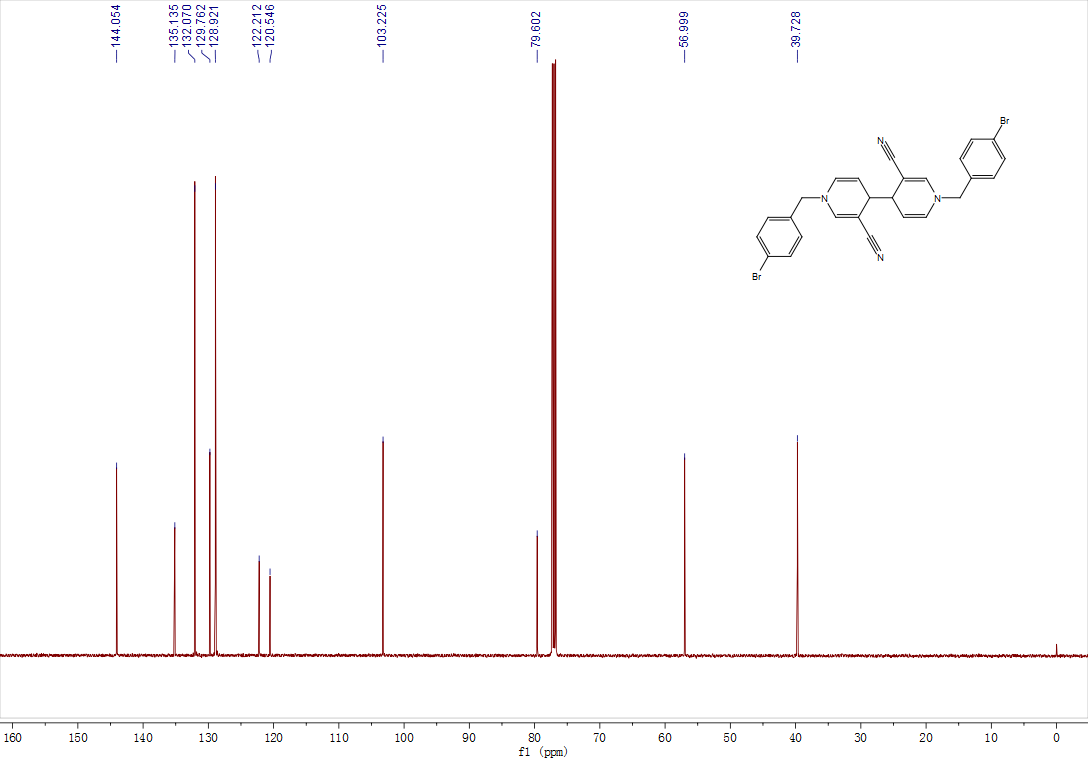


**^1^H-NMR of 2l (400 MHz**)


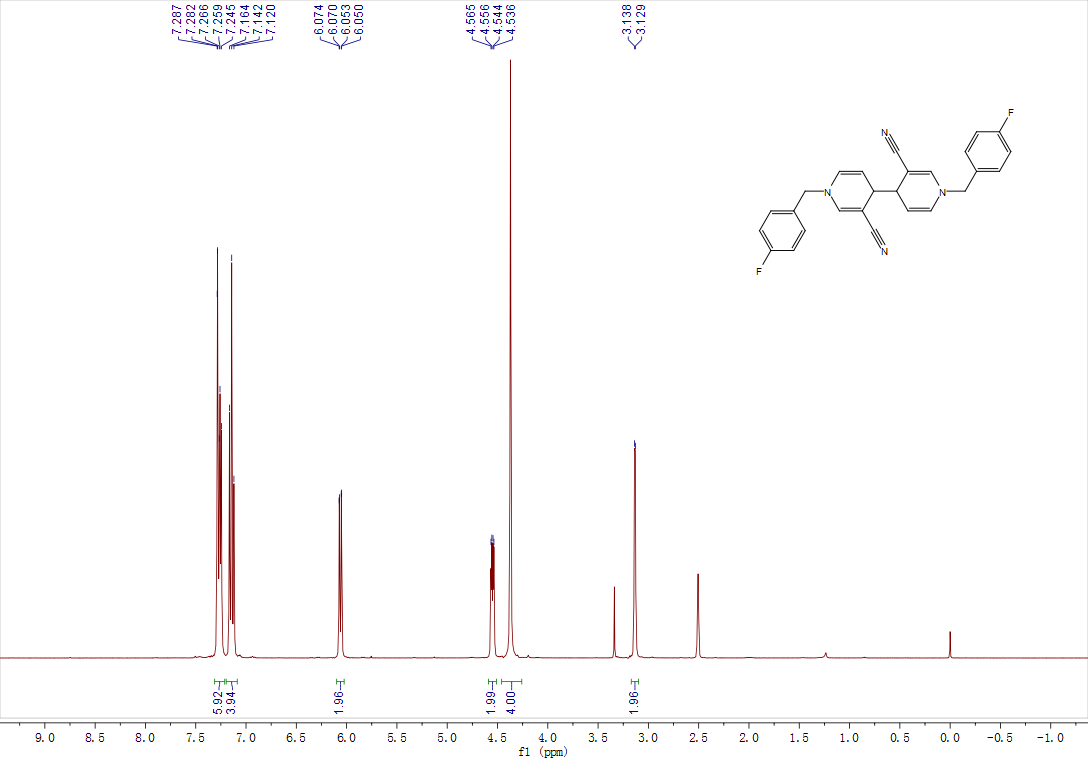


**^13^C-NMR of 2l (100 MHz**)


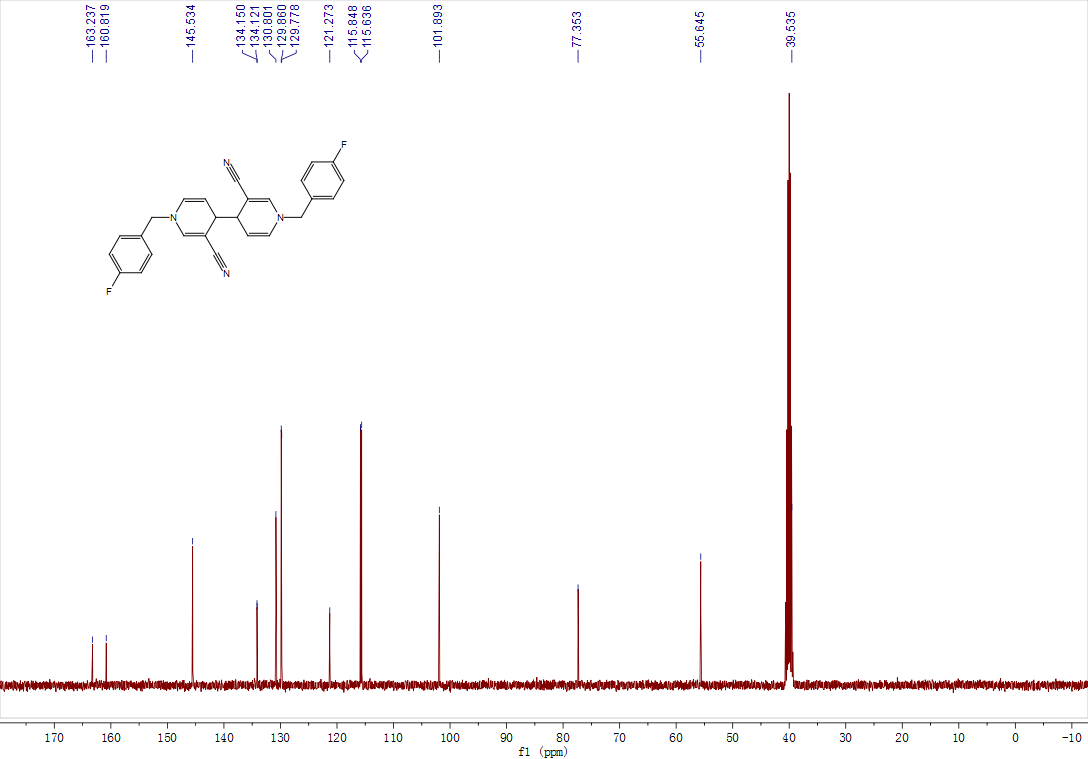


**^1^H-NMR of 2m (500 MHz**)


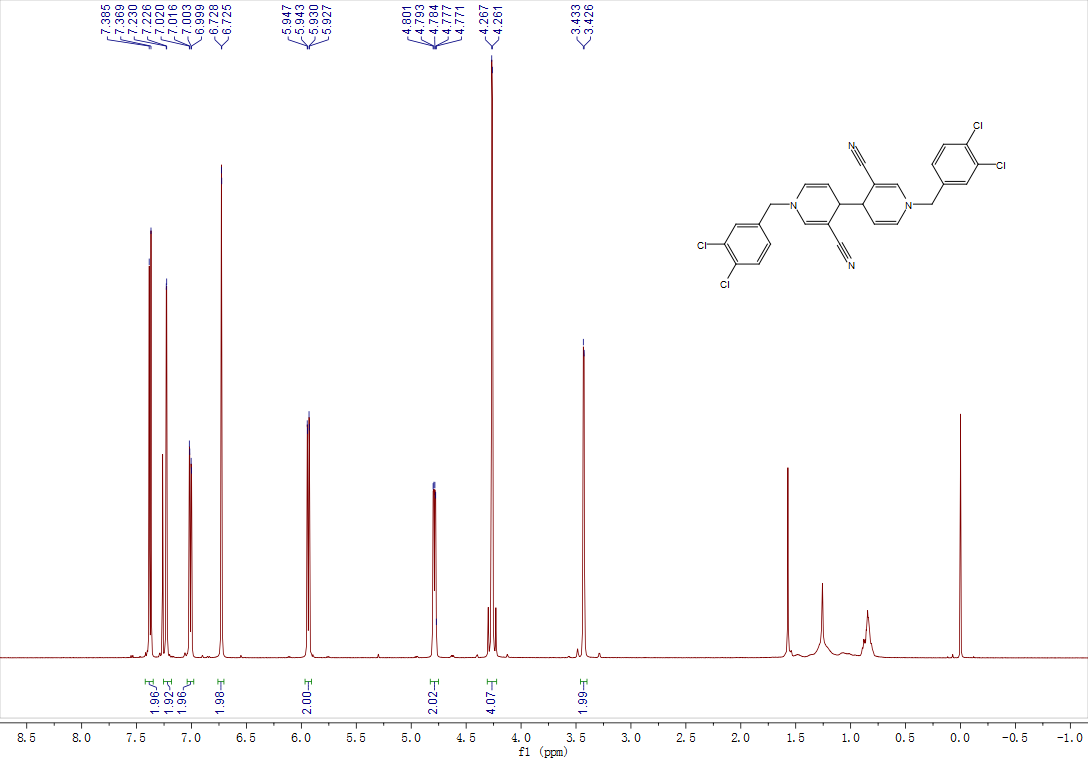


**^13^ C-NMR of 2m (125 MHz**)


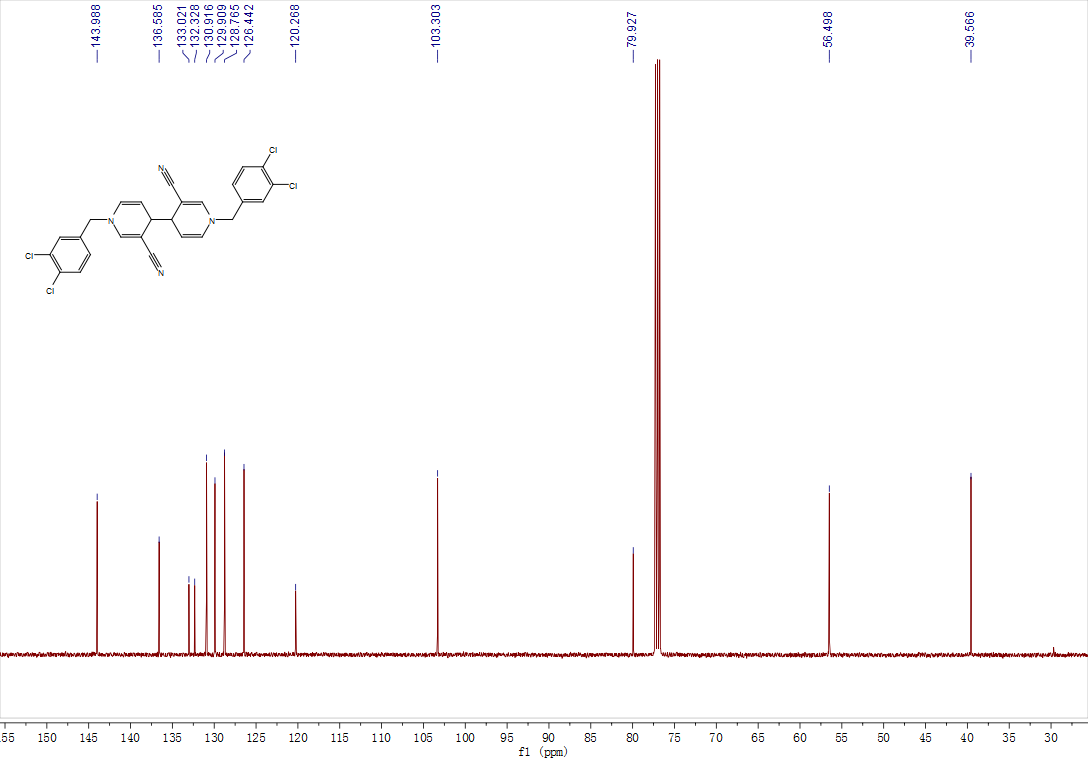


**^1^H-NMR of 2n (500 MHz**)


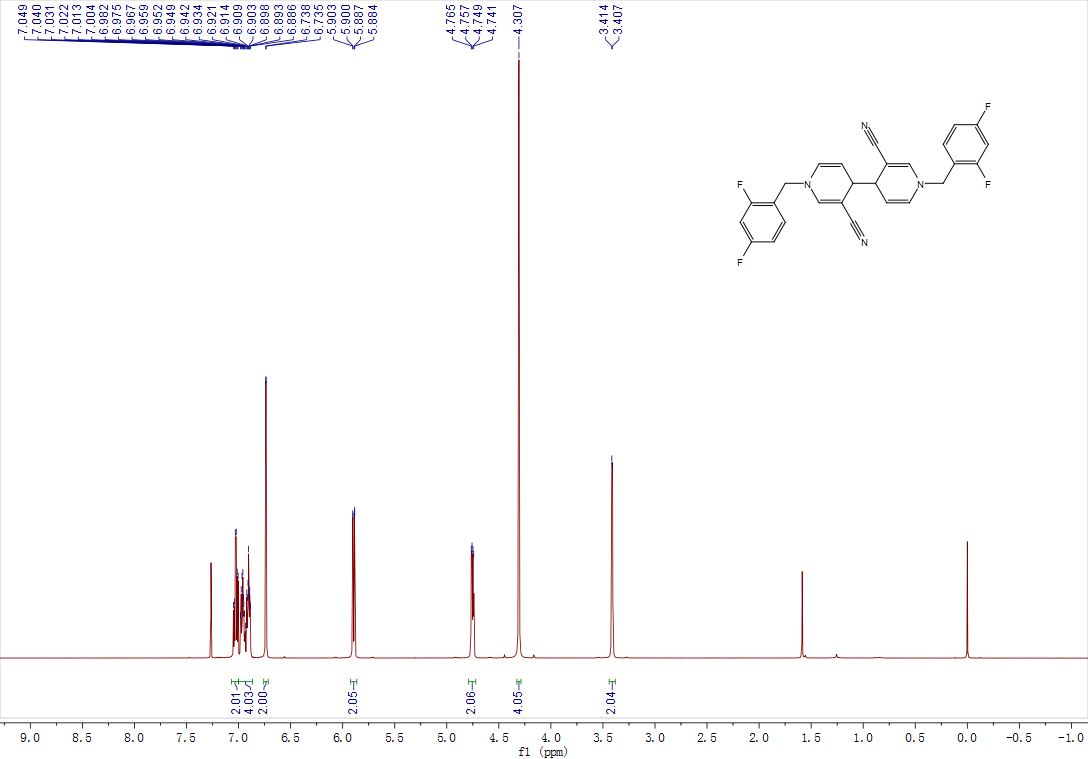


**^13^C-NMR of 2n (125 MHz**)


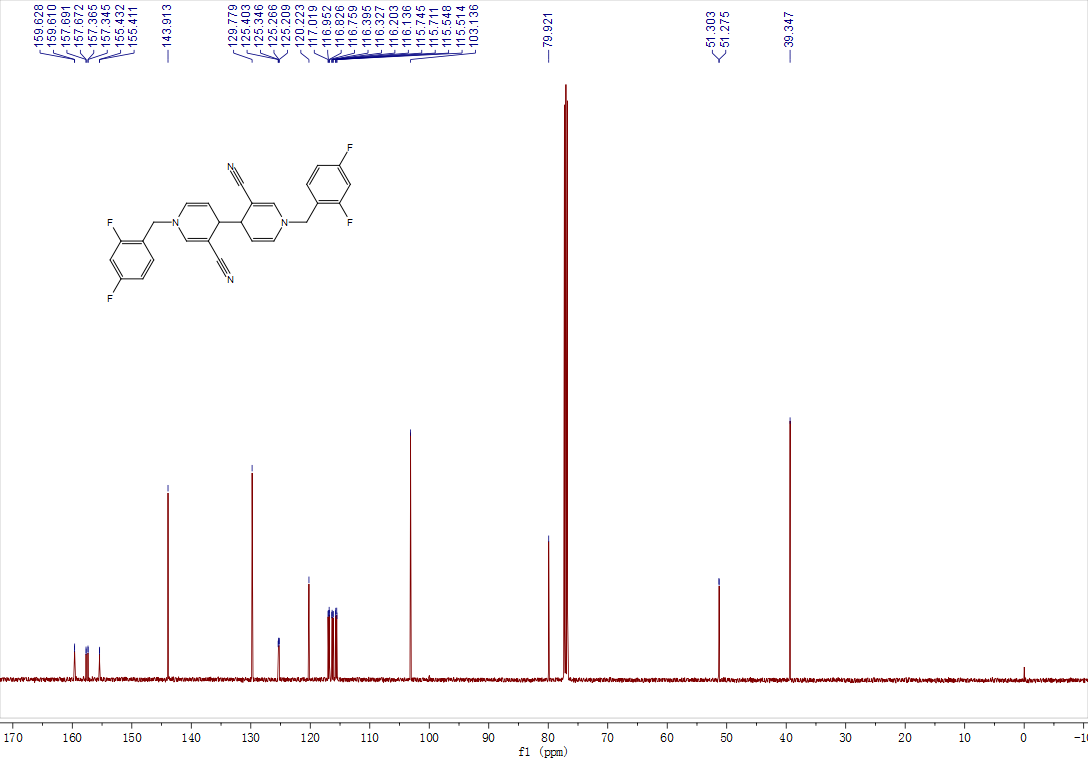


**^1^H-NMR of 2o (500 MHz**)


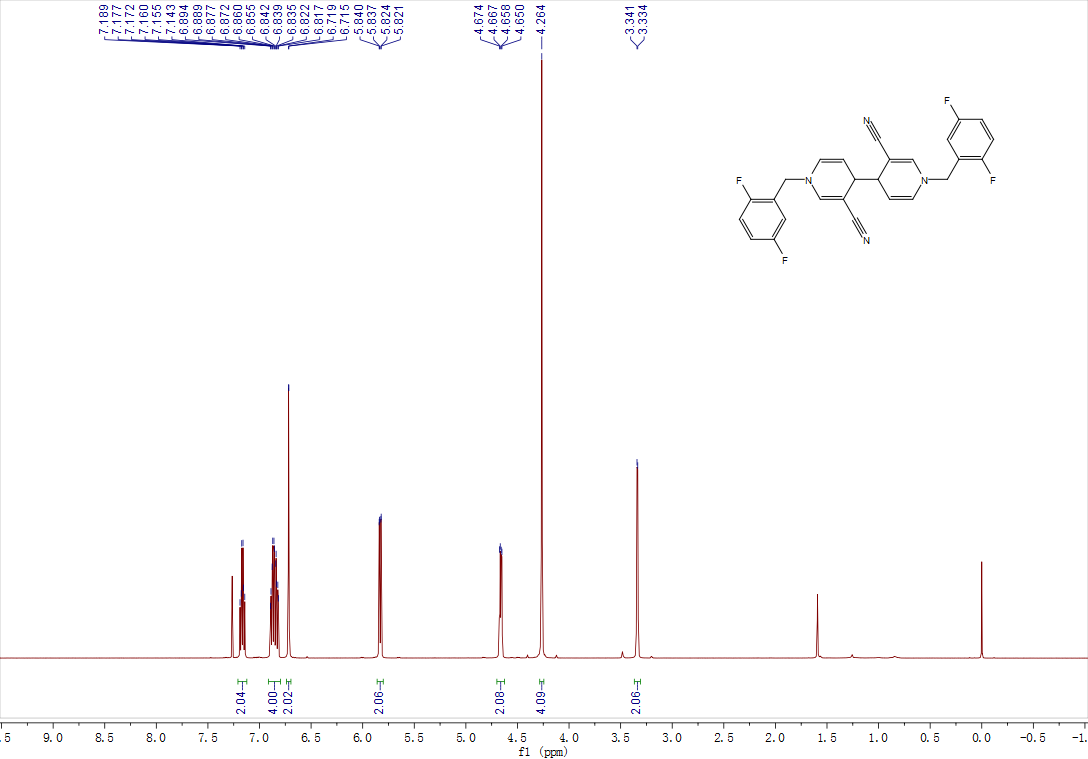


**^13^C-NMR of 2o (125 MHz**)


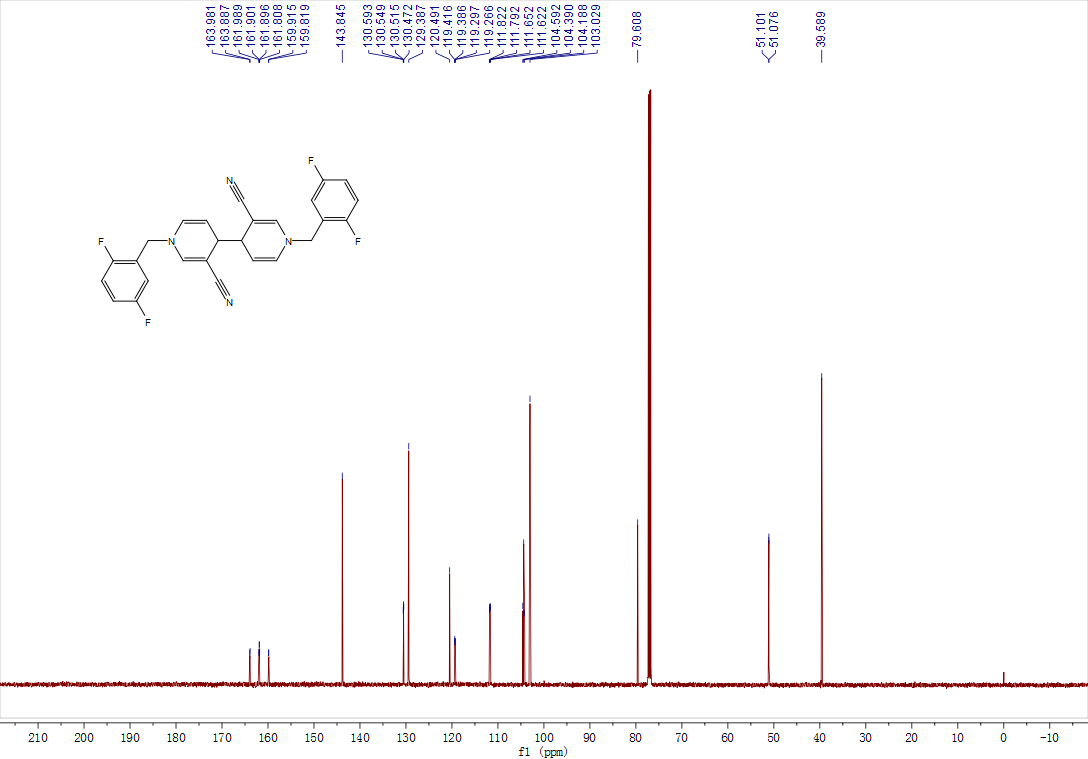


**^1^H-NMR of 2p (500 MHz**)


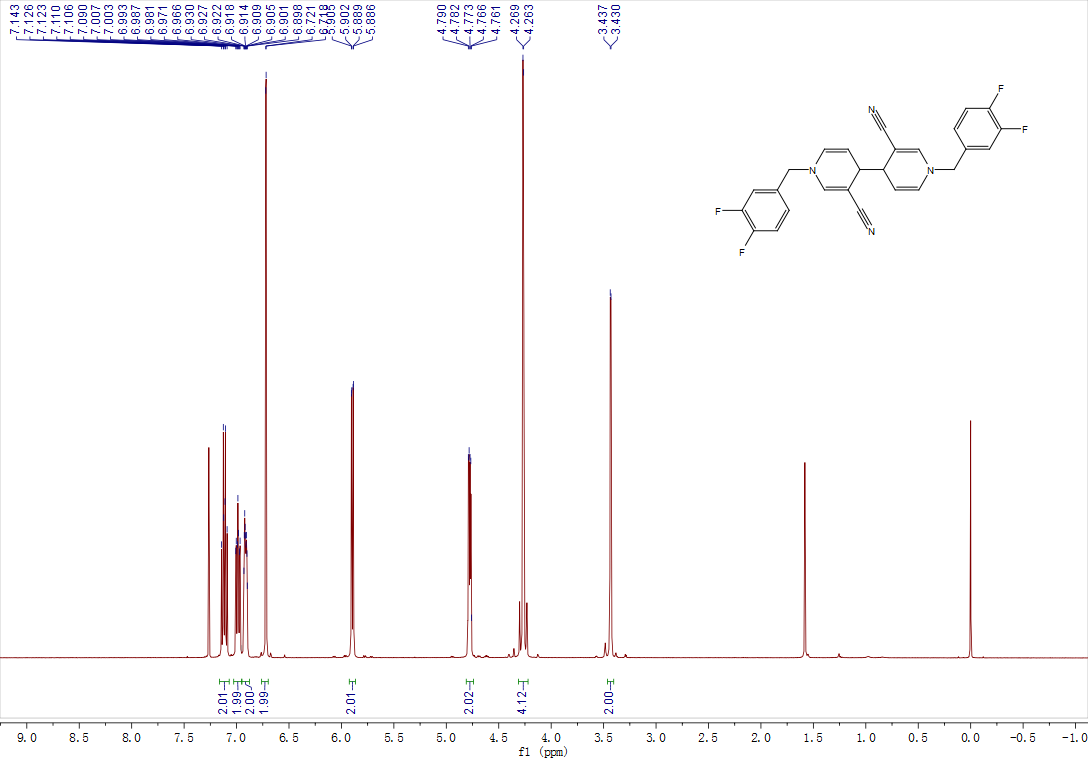


**^13^C-NMR of 2p (125 MHz**)


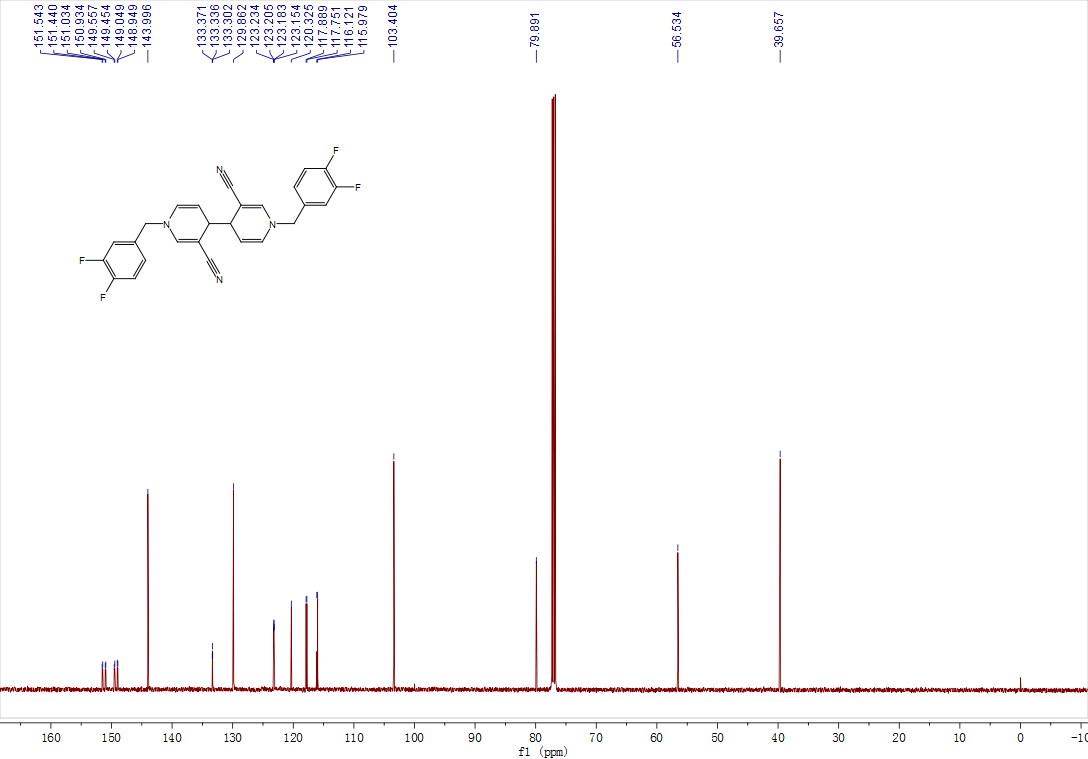


**^1^H-NMR of 2q (500 MHz**)


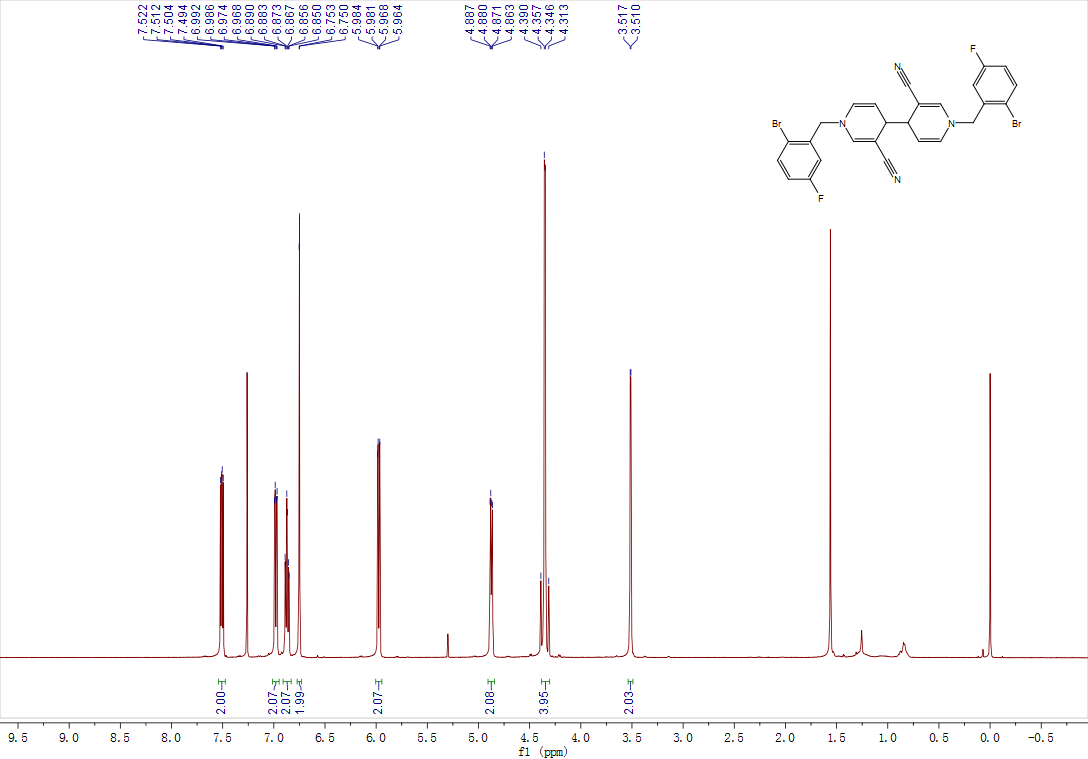


**^13^C-NMR of 2q (125 MHz**)


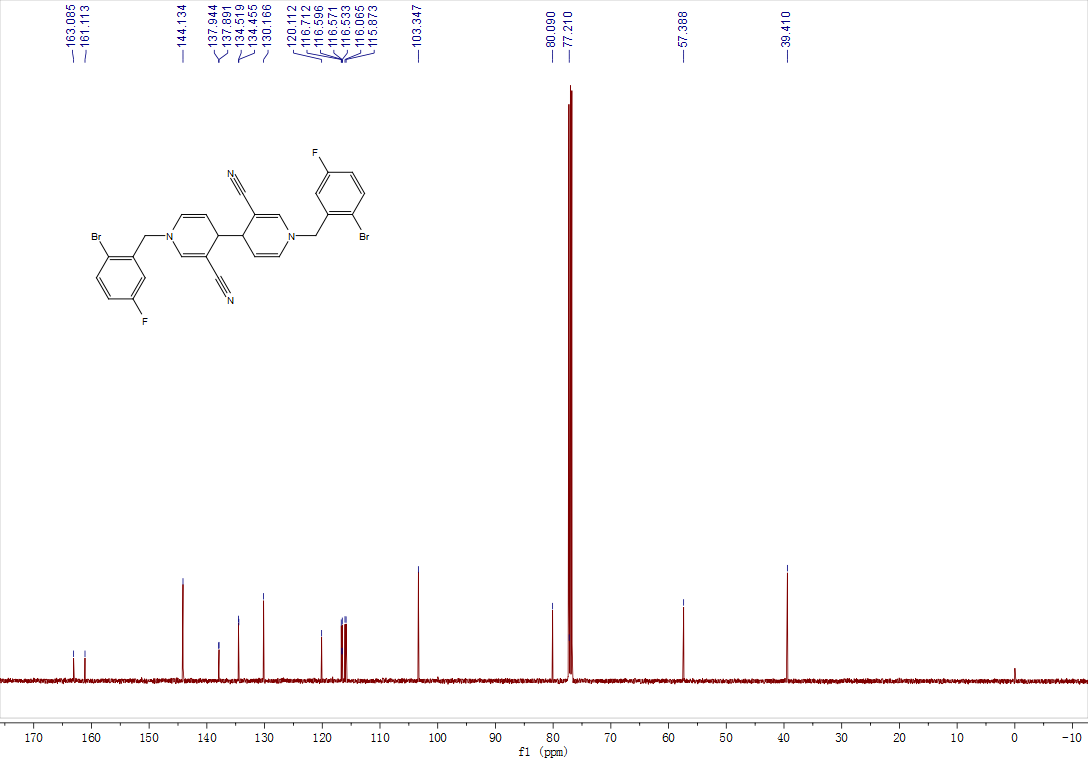


**^1^H-NMR of 2r (500 MHz**)


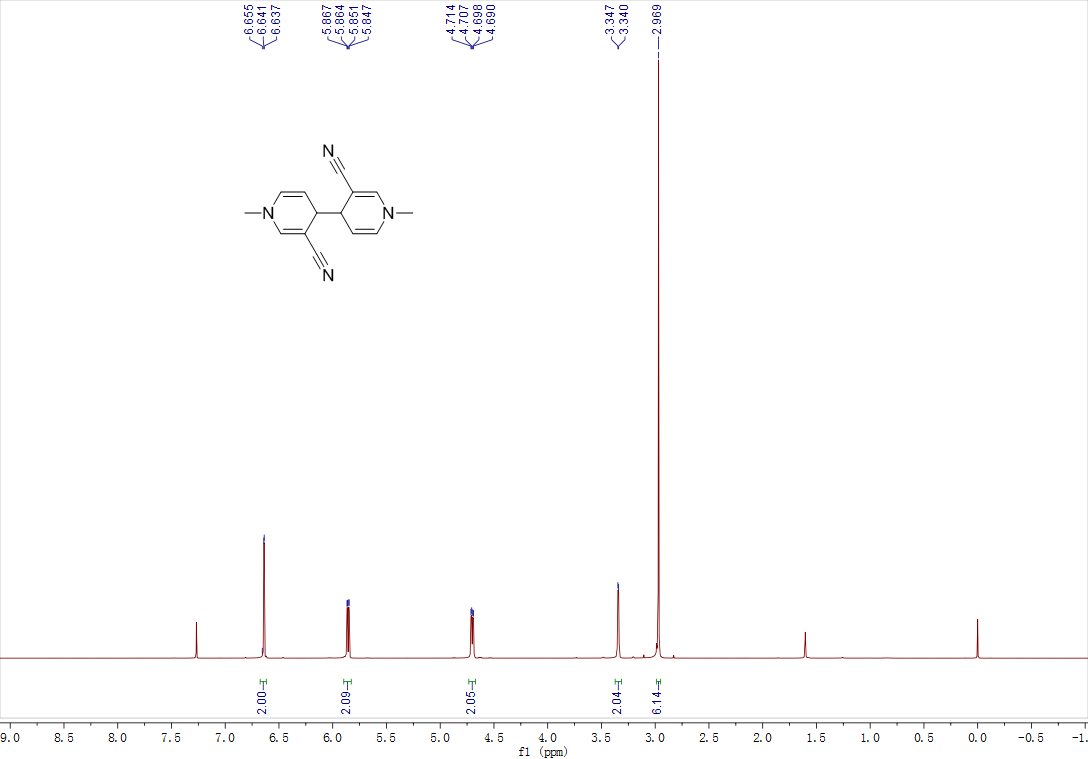


**^13^C-NMR of 2r (125 MHz**)


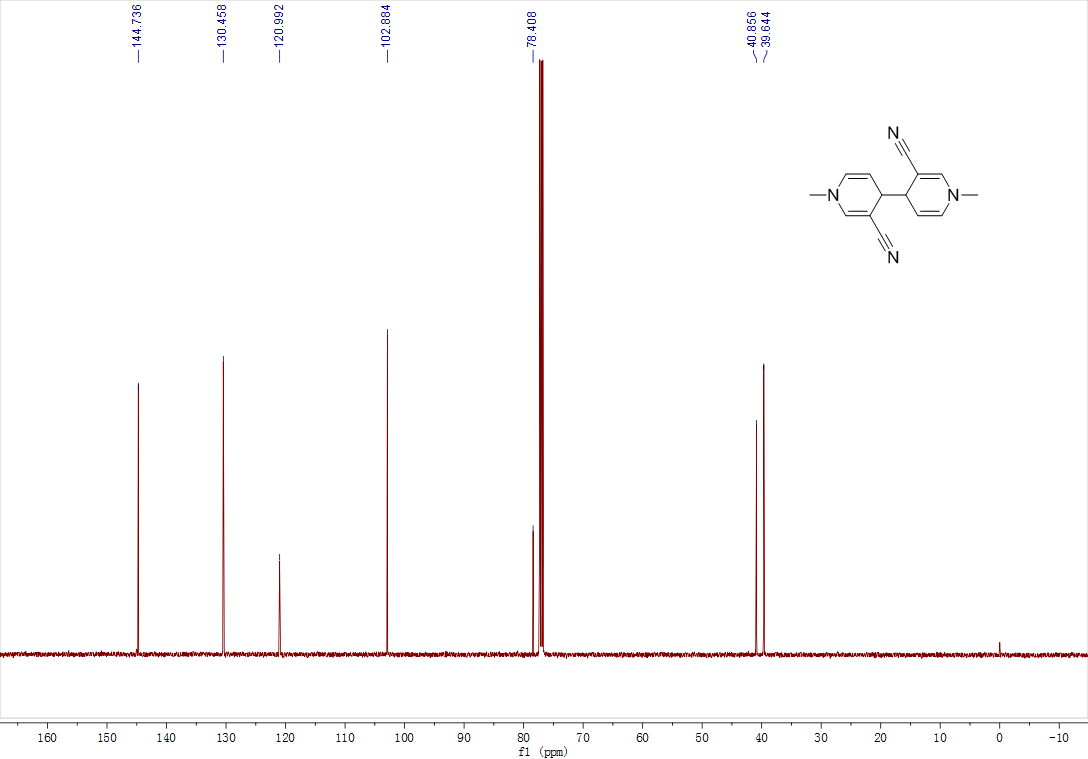


**^1^H-NMR of 2s (500 MHz**)


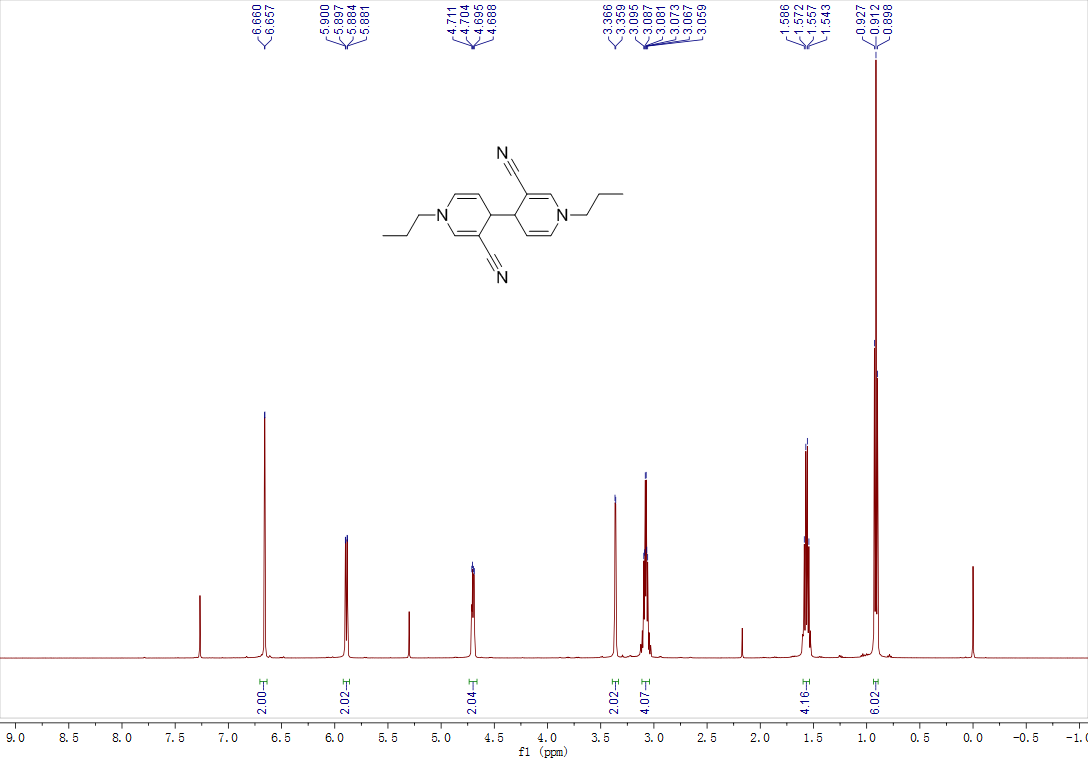


**^13^C-NMR of 2s (125 MHz**)


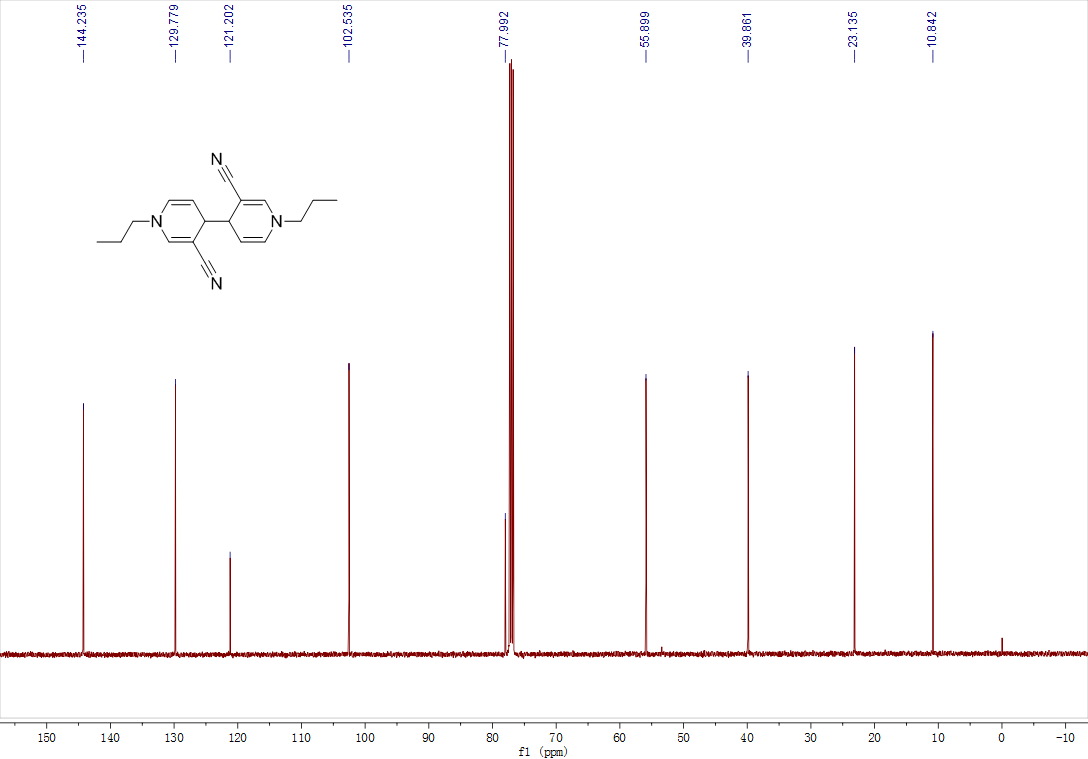


**^1^H-NMR of 2t (500 MHz**)


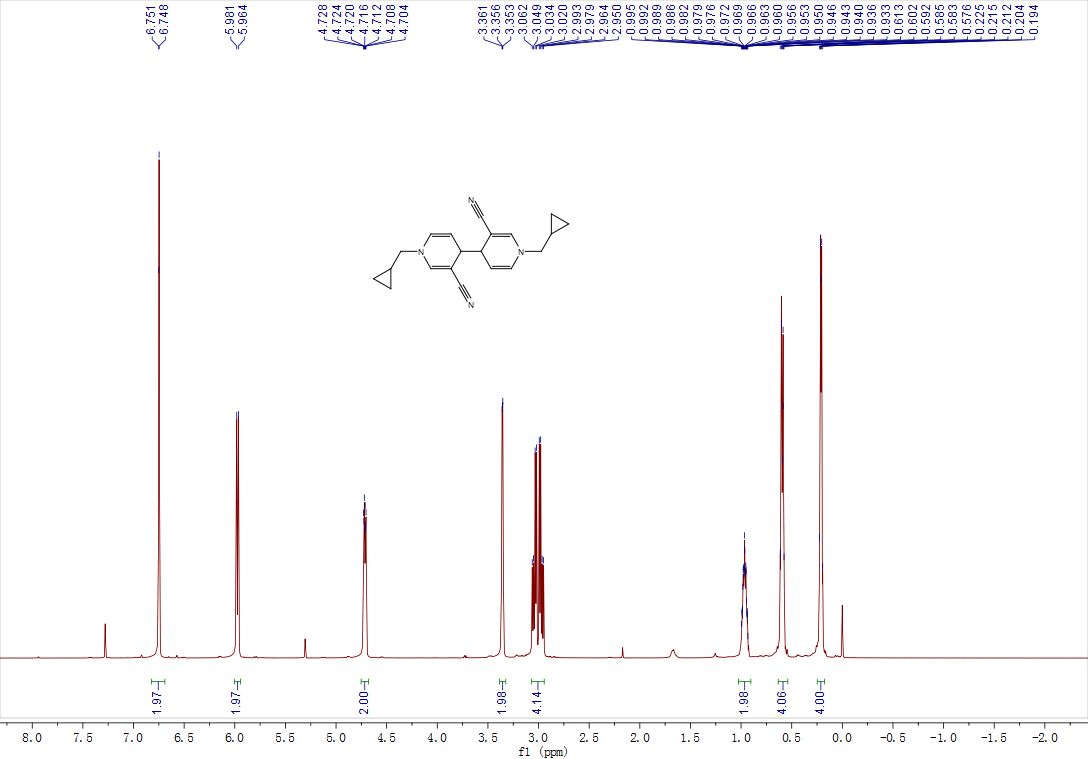


**^13^C-NMR of 2t (125 MHz**)


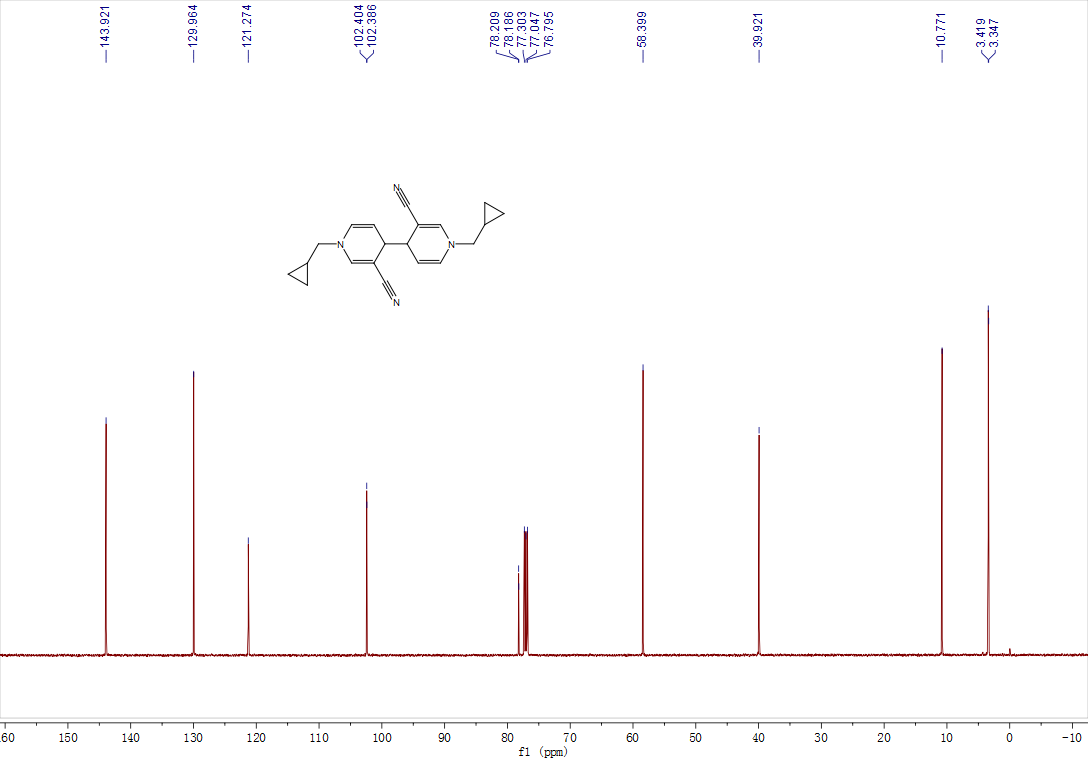


**^1^H-NMR of 3a (400 MHz**)


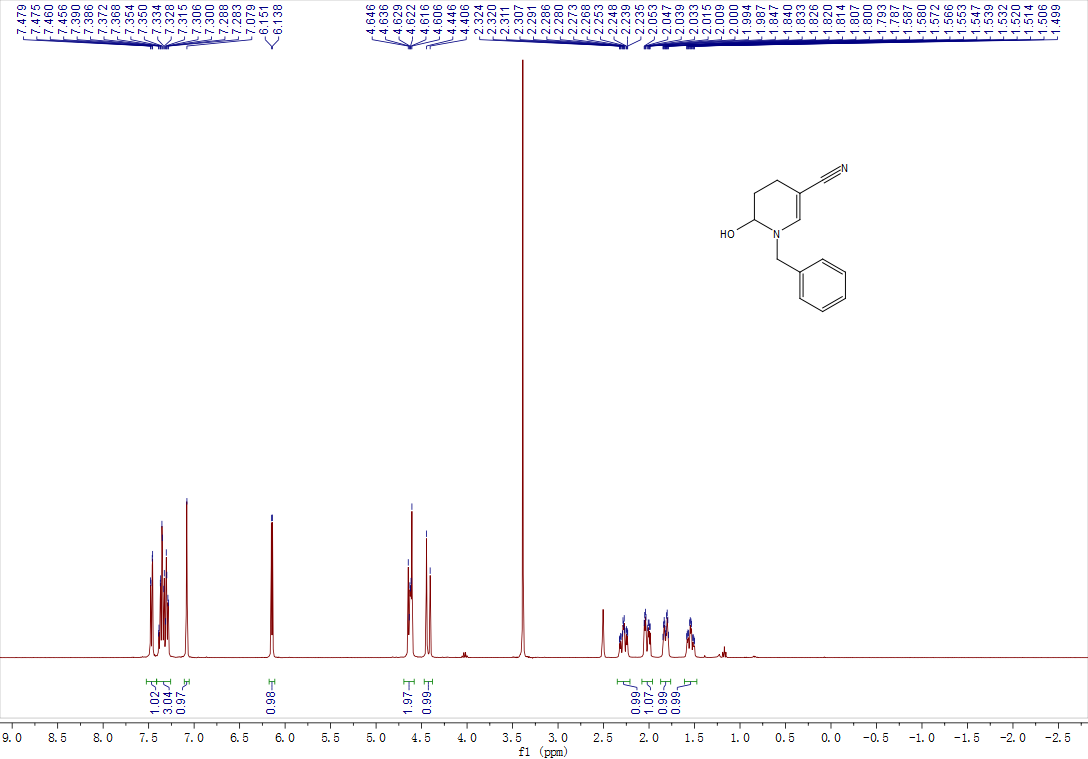


**^13^C-NMR of 3a (100 MHz**)


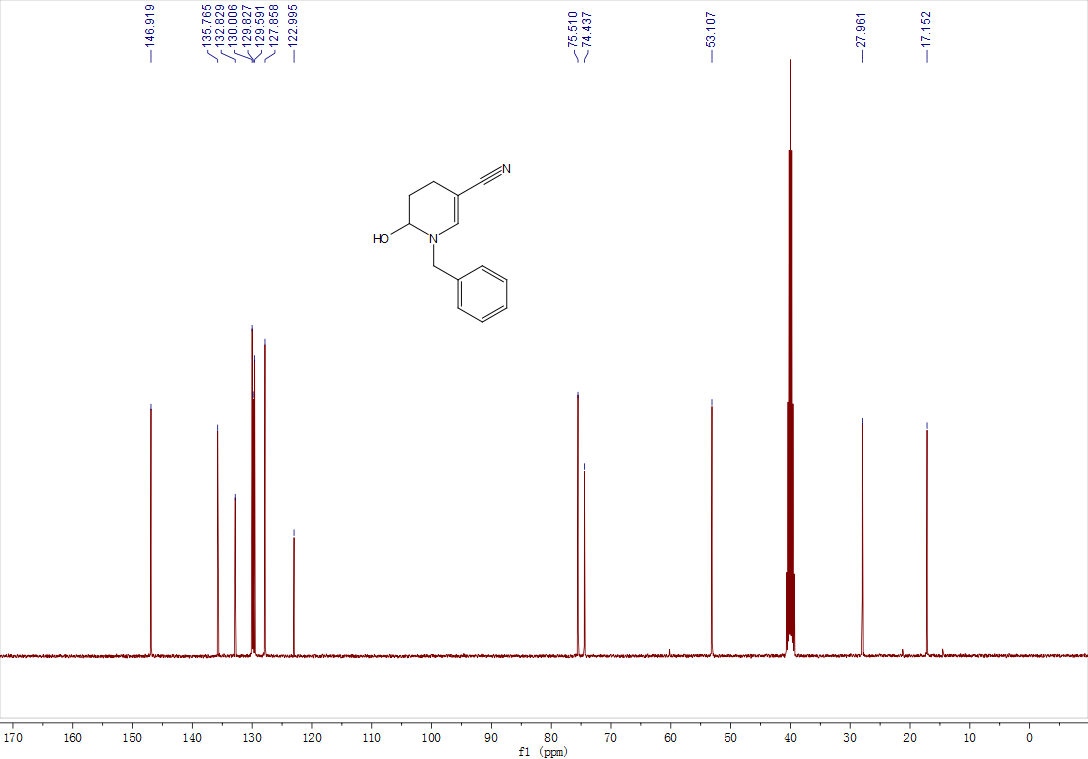

Supplement: Supplementary file 1 [file Table_1.DOCX]
